# Queuosine tRNA modification regulates translational adaptation and virulence of *Leishmania mexicana*

**DOI:** 10.1371/journal.pbio.3003887

**Published:** 2026-07-17

**Authors:** Bankatesh Kumar, Julie Kovářová, Michala Boudová, Sneha Kulkarni, Thalia Pacheco Fernandez, Abhay Satoskar, Zdeněk Paris

**Affiliations:** 1 Institute of Parasitology, Biology Centre, Czech Academy of Sciences, České Budějovice, Czech Republic; 2 Faculty of Science, University of South Bohemia, České Budějovice, Czech Republic; 3 Department of Pathology and Microbiology, The Ohio State University, Columbus, Ohio, United States of America; University of Melbourne, AUSTRALIA

## Abstract

The complex life cycle of the human parasite *Leishmania mexicana* requires rapid translational adaptation for survival in two distinct environments: the insect vector and the mammalian host. These protists lack conventional transcriptional control due to their unusual genome organization. Consequently, tRNA modifications may represent an additional mechanism for post-transcriptional regulation of gene expression. One such modification is queuosine (Q), which is incorporated at the anticodon wobble position 34 of specific tRNAs. Here, we demonstrate that Q-tRNA levels increase substantially during *Leishmania* differentiation from the insect stage to the mammalian-infective stage, implying an important role for virulence. Hence, we generated mutant cells lacking the enzyme responsible for Q incorporation, tRNA-guanine transglycosylase (TGT), which exhibited substantial changes in the proteome during differentiation in vitro. Specifically, downregulated proteins were enriched in NAU codons, whereas upregulated proteins predominantly contained NAC codons. Although LmxTGT knockout parasites exhibited normal growth and differentiation in vitro, they demonstrated impaired survival within macrophages and reduced pathogenicity in mice, highlighting the role of the Q-tRNAs under stress conditions. To our knowledge, we present here the first direct evidence that queuosine tRNA modification controls the infectivity of *Leishmania* via codon-biased translation. To date, gene expression regulation in *Leishmania* and other trypanosomatids has been attributed mostly to RNA stability and processing; however, our findings demonstrate that tRNA modifications also play a key regulatory role. Specifically, the Q-tRNA modification provides a novel layer of gene expression regulation, maintaining translational balance and supporting the parasite’s ability to adapt to changing environments, and contributing to *Leishmania* virulence.

## Abstract 2

Regulation of gene expression in the protozoan parasite Leishmania mexicana is known to be largely post-transcriptional, but the control of these processes is still not fully understood. This study shows that modification of tRNAs by quenosine influences codon-biased translation and contributes to Leishmania virulence.

## Introduction

*Leishmania mexicana* (*L. mexicana*) is a kinetoplastid protozoan parasite and the primary cause of cutaneous leishmaniasis in North and Central America [1,2]. Leishmaniasis is a significant public health issue due to its high morbidity, causing skin lesions and long-term disability [1–3]. Despite this, there are no approved vaccines for humans, and existing treatments often carry severe toxicity risks and can lead to parasite resistance [4–6]. *Leishmania* parasites are transmitted to mammalian skin tissue during a blood meal via saliva of an infected sand fly. As a result, metacyclic promastigotes, which represent the infectious stage of *L. mexicana*, are rapidly phagocytosed by macrophages recruited to the site of the sand fly bite. Within the phagolysosomal compartment of these host cells, the parasites differentiate into the amastigote stage and proliferate intracellularly. During a subsequent blood meal, infected macrophages are taken up by another sand fly, continuing the transmission cycle [7]. The progression of *Leishmania* life cycle involves a series of differentiation processes, including rapid remodeling of cellular architecture and physiological properties that enable adaptation to environmental changes [8,9]. *Leishmania* and other trypanosomatids, such as *Trypanosoma brucei* and *Trypanosoma cruzi*, share distinctive mechanisms for regulation of gene expression [9]. Unlike many eukaryotes, functionally unrelated genes in these parasites are organized into large, polycistronic transcription units that lack conventional promoter sequences. Transcription by RNA polymerase II begins in strand-switch regions, producing polycistronic pre-mRNAs [10]. These are processed into mature mRNAs through *trans-*splicing, which appends a splice leader (SL) RNA at the 5′ end and polyadenylation at the 3′ end. Consequently, gene expression regulation is largely post-transcriptional, involving mRNA stability, processing, and translation efficiency mediated, for instance, by 3′ UTR sequences [11–13]; however, control of these processes is still not fully understood.

**Fig 1:**
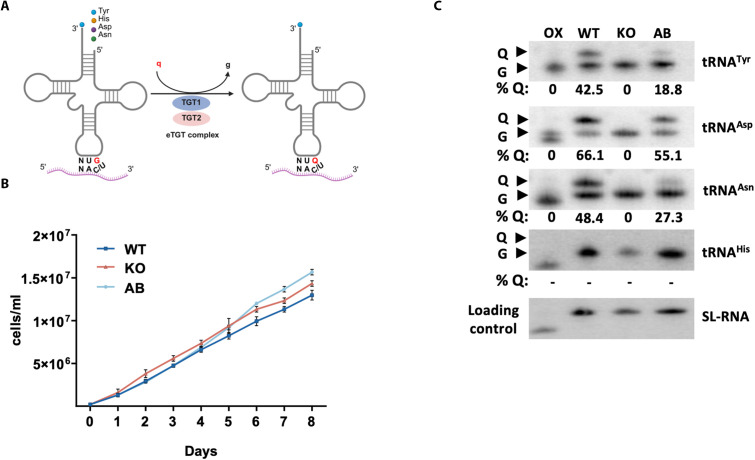
LmxTGT2 is necessary for Q-tRNA formation. **(A)** Schematic representation of the formation of queuosine (Q) tRNA modification in eukaryotes by the heterodimeric tRNA-guanine transglycosylase (TGT) complex, which catalyzes the replacement of guanine (g) with queuine (q) in four specific tRNAs (Created in BioRender. Paris, Z. (2026) https://BioRender.com/fyga397). **(B)** Growth curves of wild-type (WT), LmxTGT2-KO (KO), and add-back (AB) promastigote cells exhibit no differences under standard exponential growth conditions with regular dilution (*n* = 3). **(C)** Northern hybridization showing the effect of LmxTGT2 gene knock-out on Q-modified tRNA levels. Total RNA from WT, LmxTGT2-KO, and AB cells was separated on an APB affinity gel and hybridized with ³²P-labeled tRNA specific probes. As a result, Q-tRNAs migrate slower on the gel, leading to separation of modified (Q) and unmodified (G) tRNAs. Periodate oxidation (OX), which eliminates the mobility shift by oxidizing *cis*-diols (queuosine as well as terminal 3′ ribose), serves as a negative control. The SL RNA is used as a loading control. The numbers below the blot indicate the percentage of Q-modified tRNA, calculated by dividing the intensity of the Q band by the sum of the Q and G bands and multiplying by 100. The results are representative of three independent experiments. The underlying data can be found in S1 Data.

Another potential mechanism for fine-tuning of gene expression at the post-transcriptional level involves tRNA modifications that affect pairing at the wobble position (position 34). Depending on their chemical properties, these modifications can alter codon recognition potential and directly influence translational efficiency and fidelity, thereby affecting overall gene expression [14]. One such modified nucleoside is queuosine (Q), a hypermodified analogue of guanosine (G), which is found in tRNAs with the GUN anticodon sequence, including those for asparagine (Asn), aspartic acid (Asp), histidine (His), and tyrosine (Tyr) [15]. Q is present in all domains of life, but although bacteria can synthesize it de novo, this biosynthetic pathway is absent in eukaryotes. Therefore, they acquire the fully modified free base queuine (q) from their diet or the gut microbiome [16,17] or salvage it in the cytosol through queuine-nucleoside hydrolase (QNG1), which mediates the recovery of queuine from queuosine-5′-monophosphate as the biological substrate [18]. In eukaryotes, the formation of Q-tRNAs involves an isoenergetic reaction catalyzed by the highly conserved heterodimeric enzyme tRNA-guanine transglycosylase (TGT) [19–21] (Fig 1A), which breaks the glycosidic bond between the base and the sugar to replace the encoded guanine at position 34 with q. The eukaryotic enzyme consists of the catalytically active subunit QTRT1 (TGT1) and the noncatalytic regulatory subunit QTRT2 (TGT2). Structural and biochemical studies in mammals demonstrate that QTRT1 alone is inactive and requires QTRT2 for functional tRNA binding and catalysis [22,23]. Similarly, in the trypanosomatid parasite *T. brucei*, both subunits are necessary for Q-tRNA formation [24]. Building on prior work in *T. brucei* and based on the functional interdependence of the eukaryotic TGT heterodimeric complex, we focused our analysis on the TGT2 subunit to investigate the consequences of Q-tRNA loss in *L. mexicana*.

Several studies point to the involvement of Q-tRNAs in processes such as cell proliferation, differentiation, or virulence [15,25,26]. The position of Q at the wobble base of the anticodon of tRNA strongly implicates its role in translation via a mechanism based on codon usage. It has been suggested that Q-tRNA plays a crucial role in translation by modulating codon-anticodon interactions. In silico-based analysis has shown that while unmodified tRNAs prefer NAC codons over NAU codons, Q-tRNAs accelerate translational speed at NAU codons [27]. Furthermore, in vivo, Q34 in cytoplasmic tRNAs was shown to regulate elongation speed, ensuring proper folding of nascent proteins and maintaining proteome integrity [17]. In addition to its cytoplasmic role [28,29], Q34 is also involved in mitochondrial translation, where it facilitates efficient NAU decoding in human mitochondria [30]. These findings are in line with our previous observations in *T. brucei*, where Q-modified tRNAs are preferentially imported into the mitochondria, playing a crucial role in organellar protein synthesis [24]. Without the Q modification, mitochondrial translation is impaired, leading to reduced activity of respiratory complexes and highlighting the importance of Q modification for organellar physiology [24]. While the predominant trend seen in mammals [29], *T. brucei* [24], and bacteria [31] suggests that Q-tRNA preferentially improves decoding of Q-dependent codons ending with U over those ending with C, a few exceptions exist. In *Drosophila,* Q-tRNA demonstrates a bias toward NAC codons [32]. Similarly, in yeast *Schizosaccharomyces pombe*, Q modification has been shown to regulate translation speed by enhancing the decoding of C-ending codons for aspartate and histidine while reducing the speed of U-ending codons for asparagine and tyrosine [33]. Moreover, the position and sequence context of the impacted codons may also be important.

All these findings underscore the regulatory potential of Q-tRNA to modulate codon usage bias and translation efficiency, which could have significant implications for the adaptability and virulence of protozoan parasites such as *L. mexicana*. Additionally, recent research in mammals links Q-tRNA to translation fidelity, with its loss associated with impaired translation elongation and changes in neuronal morphology [34]. Similarly, in human cells lacking queuine, codon usage imbalances have been observed in genes linked to differential protein expression, further emphasizing the regulatory role of Q in translation [29,33]. While Q-tRNA modification has been associated with biofilm formation and virulence in bacteria [31], highlighting its role in pathogenesis, the impact on virulence in eukaryotic pathogens remains largely unexplored.

In this study, we investigated the role of queuosine tRNA modification in *L. mexicana* and its role in parasite differentiation and virulence. Using CRISPR/Cas9, we generated LmxTGT2 knockout parasites and established that Q-tRNA modification influences codon-biased translation, specifically affecting the expression of genes enriched in NAU codons. We further examined the functional consequences of Q-tRNA depletion in both macrophage infections and an in vivo mouse model. While LmxTGT2 knockout cells exhibited normal growth and differentiation when cultivated in vitro, their ability to survive within macrophages was compromised, and they demonstrated markedly decreased virulence in mice. Our findings highlight the crucial role of queuosine tRNA modification in regulating *Leishmania* translation and consequently its virulence to the mammalian host.

## Materials and methods

### Cell culture

The three cell lines wild-type (WT), LmxTGT2 knock-out (KO), and add-back (AB) strains of *L. mexicana* (isolate MNZC/BZ/62/M379) were grown in M199 medium (Sigma-Aldrich, Cat. No. M0393-10X1L), which was supplemented with 2 μg/ml biopterin (Sigma-Aldrich, Cat No. B2517-25MG), 2 μg/ml hemin (Sigma-Aldrich, Cat No. 51280-5G), 25 mM HEPES (Applichem, Cat. No. A3268), 100 units/ml penicillin, 100 μg/ml streptomycin (Sigma-Aldrich, Cat. No. P4333-100ML), 10% fetal bovine serum (FBS) (Sigma-Aldrich, Cat. No. F7524-500ML), and 100 μg/ml hygromycin (Invitrogen, Cat. No. ant-hg-1) at 25 °C. The medium used for the cultivation of the LmxTGT2-KO cell line was also supplemented with 100 μg/ml nourseothricin (Jena Bioscience, Cat No. AB-102L) and 50 μg/ml puromycin (Invitrogen, Cat No. ant-pr-5), serving as selection markers. To determine the cell density, samples were fixed with a LeishFix solution (3.6% formaldehyde, 150 mM NaCl, 15 mM Na_3_C_6_H_5_O_7_) and cell count was determined using a Neubauer hemocytometer (Sigma-Aldrich, Cat No. BR717805-1EA). Bone marrow-derived macrophages were cultured in DMEM/F12 medium (Invitrogen, Cat. No. 10565), supplemented with 100 units/ml penicillin and 100 μg/ml streptomycin (Sigma-Aldrich, Cat. No. P4333-100ML) and 10% FBS at 37 °C (prior to infection) or 34 °C (post-infection) and 5% CO_2_.

### Generation of LmxTGT2-KO and AB cell lines

The LmxTGT2-KO cell line was generated by CRISPR/Cas9 approach using the cloning strategy described by Ishemgulova and colleagues [35]. The LmxTGT2 gene specific single guide RNA (sgRNA) (sequence: GAAGATGGTGCAGGTAAGCG│CGG) was designed using the eukaryotic pathogen CRISPR guide RNA/DNA design tool (http://grna.ctegd.uga.edu) [36], amplified by PCR using specific primers (S1 Table), and cloned into a modified pLEXSY-SAT2.1 vector (Jena Bioscience, Cat. No. EGE-274) containing a U6 promoter and a nourseothricin resistance marker. The vector was kindly provided by Prof. Vyacheslav Yurchenko (Life Science Research Centre, Faculty of Science, University of Ostrava). The repair template was PCR-amplified using corresponding oligonucleotides (S1 Table), generating a cassette containing the puromycin resistance gene flanked by 30 bp LmxTGT2 homology arms at the Cas9 cleavage sites. Ten micrograms of both the sgRNA construct and repair template were co-transfected into the *L. mexicana* Cas9-expressing cell line using the Amaxa Nucleofector program U-033. LmxTGT2 ablation was confirmed by PCR on genomic DNA using specific primers (S1 Table). The add-back cell line was generated by expressing a Cas9-resistant version of the LmxTGT2 gene cloned in a modified pLEXSY vector, which contains UTRs of the A600 gene, constitutively expressed throughout all parasite life stages.

### Northern blot analysis

Total RNA was isolated by phenol–chloroform extraction as previously described [37]. For aminophenylboronic acid (APB) affinity electrophoresis, total RNA samples were deacylated by incubating them in 100 mM Tris (pH 9.0) for 30 min. To create an oxidation control, RNA was further treated with 2 mM NaIO_4_ in 50 mM Tris (pH 5.0) in the dark for 2 hours at 37 °C, preventing APB binding by modifying the ribose hydroxyl groups. The oxidation reaction was subsequently quenched with 2.5 mM glucose before use. A 10 ml gel mixture (8 M urea, 8% polyacrylamide) was supplemented with 25 mg of APB (Sigma-Aldrich) before polymerization [38]. Electrophoresis of APB gels was conducted for ~6 hours at 75 V and 4 °C, followed by electroblotting onto Zeta-Probe membranes (Biorad)under manufacturer’s transfer conditions. The membrane was then UV-cross-linked for 1 min. For hybridization, γ-32P ATP-labeled oligonucleotide probes were prepared using T4 Polynucleotide Kinase (NEB, Cat. No. M0201S) and purified using a Sephadex G-25 column. The probes were denatured at 98 °C for 5 min before being added to the hybridization buffer (5× SSC, 20 mM phosphate buffer, 7% SDS, 1× Denhardt’s solution, and 1 mg/ml salmon sperm DNA). The membrane was hybridized at 48 °C overnight, then washed twice at 48 °C (Wash I: 3× SSC, 5% SDS, 25 mM NaH_2_PO_4_, 10× Denhardt’s solution; Wash II: 1× SSC, 1% SDS). Hybridized membranes were exposed overnight to a phosphor-imager screen (GE Healthcare) and analyzed using a Typhoon FLA 9000 scanner with ImageQuant TL software (GE Healthcare). Probes utilized for Northern hybridization are listed in S1 Table.

### *In vitro* differentiation

To induce differentiation of *L. mexicana* from the procyclic promastigote stage into infective metacyclic forms, cultures were initiated at a density of 2 × 10^5^ cells/ml and maintained in M199 medium (pH 7) supplemented with 10% FBS for 10 days without dilution. Axenic amastigote forms were obtained by transferring the metacyclic culture into SDM medium containing 20% FBS and incubating at 32 °C for an additional four days [39].

### qPCR

To assess stage-specific gene expression by quantitative reverse transcription PCR (qPCR), RNA was harvested on days 0, 8, 10, 13, and 17 of differentiation. Reverse transcription was performed using the QuantiTect Reverse Transcription Kit (Qiagen, Cat. No. 205311). cDNA was then used for qPCR with 60S primers as a control, PfrD1 primers, Sherp primers, and Amastin primers (S1 Table). Reactions were carried out using Power SYBR Green PCR Master Mix (Thermo Fisher Scientific, Cat. No. 4367659) according to the manufacturer’s instructions and executed on the QuantStudio 6 PCR system (Thermo Fisher Scientific).

### Macrophage isolation and *in vitro* infection

Bone marrow-derived macrophages were isolated from BALB/c mice as described previously [40]. Murine macrophages were infected with *L. mexicana* parasites following a previously described protocol, with minor modifications [40]. Prior to infection, differentiated metacyclic *L. mexicana* were labeled with Cell Tracker^TM^ Orange CMRA dye (Invitrogen, Cat No. C34551) as described by Michael *and colleagues* [41]. Macrophages were primed with IFN-γ (5 ng/ml; Invitrogen, Cat. No. PMC4031) 24 hours prior to infection and subsequently activated with a combination of IFN-γ (5 ng/ml) and LPS (10 ng/ml; Sigma-Aldrich, Cat. No. L2630-10MG). Subsequently, macrophage cultures were exposed to parasites at a 1:7 ratio in a final volume of 2 ml for 4 hours at 34 °C, 5% CO2. The noninternalized parasites were removed with four successive washes with 1x PBS and subsequently replaced with fresh medium. This point was considered the initial time of infection (day 0). Cultures of infected macrophages were incubated for up to 5 days under the same conditions. To assess infection rates, cells were seeded onto coverslips placed in 6-well plates. At designated time points (days: 0, 1, 2, 3, and 5), coverslips were removed, and cells were fixed and permeabilized with ice-cold methanol for 10 min. Following fixation, cells were washed three times with 1x PBS to remove residual methanol. Subsequently, cells were stained with DAPI (Thermo Fisher Scientific, Cat No. P36941). Infection rates were determined by counting 100 cells per group under a fluorescence microscope, identifying nuclei stained with DAPI and parasites labeled with CMRA dye.

### Dual luciferase reporter constructs design and assay

Renilla and Firefly luciferases were encoded within a single open reading frame and expressed as an in-frame fusion protein from one mRNA. While the Renilla luciferase (Rluc) sequence remained unaltered as an internal control, the cognate codons of Q-tRNAs in Firefly luciferase (Fluc) were either left unchanged (50% NAC, 50% NAU) or modified to exclusively C-ending (100% NAC) or U-ending (100% NAU) codons. These two recoded constructs were synthesized by Eurofins Genomics. All three reporter constructs were cloned into the pLEXSY vector under the regulation of the A600 UTRs and introduced into *L. mexicana* WT and LmxTGT2-KO cell lines via electroporation. Positive clones were selected using puromycin. The reporter assay was performed using Promega Dual Luciferase kit, as per manufacturer’s protocol. Briefly, 2 × 10⁴ cells were washed with PBS and resuspended in Passive Lysis Buffer (PLB) in a 96-well transparent flat-bottom plate. The plate was incubated at room temperature for 15 min with continuous shaking. Firefly luciferase activity (FLuc) was measured following the addition of the LARII substrate using a Tecan Spark luminometer. Subsequently, the Stop and Glo reagent, which quenches Firefly luminescence while activating Renilla luminescence, was added, and RLuc activity was recorded. The FLuc/RLuc ratio was calculated, and values were normalized to the control construct (50% NAC + 50% NAU).

### Proteomics

WT, LmxTGT2-KO, and AB cells were collected at distinct differentiation time points (days: 0, 8, 10, 13, and 17) and analyzed at the CEITEC Proteomics Facility, Brno, Czech Republic. Briefly, proteins extracted in SDT buffer were processed using filter-aided sample preparation as described elsewhere [42]. The resulting peptides were analyzed by liquid chromatography–tandem mass spectrometry (LC-MS/MS) using the nanoElute system (Bruker) coupled to the timsTOF Pro 2 spectrometer (Bruker). Prior to LC separation, tryptic digests were online concentrated and desalted using a trapping column (Acclaim PepMap 100 C18, Thermo Fisher Scientific) and eluted onto an analytical column (Aurora C18, Ion Opticks) via a 90-min linear gradient. MS/MS data were acquired in data-independent acquisition (DIA) mode with a precursor isolation range of m/z 400–1,012. DIA data were processed using DIA-NN (version 1.8.1) [43] in library-free mode against the UniProtKB *L. mexicana* proteome database (UP000007259). Protein MaxLFQ intensities were further processed in a containerized computational environment (OmicsWorkflows, version 4.7.7a), following standard preprocessing, log2 transformation, and differential expression analysis using LIMMA. Mass spectrometry proteomics data have been deposited in the ProteomeXchange Consortium via PRIDE [44] partner repository with the dataset identifier PXD061534.

### Proteomic data analysis and GO enrichment methodology

Proteomic data analysis was performed using R for visualization, including UpSet plots, principal component analysis (PCA), and Gene Ontology (GO) term plots. Differentially expressed proteins were identified by comparing LmxTGT2-KO with WT and AB across multiple time points, focusing on those consistently up- or downregulated during differentiation. GO enrichment analysis, conducted using TriTrypDB (https://tritrypdb.org), assessed the functional impact of these proteins by mapping GO terms to relevant biological processes.

### Determination of codon bias in proteomics data

To evaluate the impact of codon bias on protein expression, proteins significantly up- or downregulated in the proteomics data were analyzed for codon usage of tyrosine (Tyr), asparagine (Asn), aspartate (Asp), and histidine (His). The corresponding mRNA sequences were retrieved from the NCBI database using UniProt IDs. For each coding sequence (CDS), the number of Q-containing codons for each amino acid was calculated as the “observed codon frequency”. The Z-score for codon usage was determined using the formula: z-score of codon = (Observed frequency of codon − Expected frequency of codons)/ Standard deviation of observed frequency of codon, where the expected frequency represents the genome-wide average frequency of the respective codon. Genome codon frequencies were obtained from DNA Hive [45]. Analysis was performed in RStudio (version 4.3.3), with ggplot2 used for data visualization.

### Ethical statement

Mice were housed and handled in the animal facility of the Biology Centre in accordance with institutional guidelines and Czech legislation (Act No. 246/1992 and 359/2012). All experiments were approved by animal ethics committee and conducted under permit No. PP 56_2022P from the Czech Ministry of Environment.

### Mice infection

Six-week-old BALB/c female mice were subcutaneously inoculated in the right ear with 1 × 10⁶ of WT, LmxTGT2-KO, and AB strains of metacyclic *L. mexicana*. M199 medium, serving as a blank control, was injected into the ears of the control group mice. Six mice per group were used for the infection study. Ear thickness was measured using a Teclock standard-type thickness gauge, with a range of 0–20 mm and a scale interval of 0.01 mm. Lesion size was monitored weekly. To determine the lesion thickness, the thickness of the uninfected ear was subtracted from the thickness of the infected ear for each experimental animal.

### Antibody ELISA

To prepare the antigen, *L. mexicana* parasites were washed three times with 1× PBS and subjected to three freeze-thaw cycles, alternating between liquid nitrogen and a 37 °C water bath. The protein concentration was determined using a BCA assay (Pierce BCA Protein Assay Kit, Thermo Fisher Scientific). Medium-binding 96-well plates were coated with 5 μg/ml of freeze-thawed *L. mexicana* antigen and incubated overnight at 4 °C. The following day, plates were washed with PBS-Tween (0.05% Tween-20 in 1× PBS, pH 7.4) and blocked with 5% milk in PBS-Tween for 1 hour at 37 °C. Blood samples were collected from infected BALB/c mice via submandibular vein bleeding every two weeks. Serum was separated by centrifugation and used for the antibody ELISA assay. After washing with PBS-Tween, serum samples were added in duplicate to the first well, followed by serial dilution across the plate. The plates were incubated for 2 hours at 37 °C, followed by washing. The plates were then incubated with HRP-conjugated antibodies IgG1 (BD Biosciences, Cat#559626, clone X56) or IgG2a (BD Biosciences, Cat#553391, clone R19-15) at a final concentration of 0.2 μl in 1 ml of PBS-FBS (3:1) for 1 hour at 37 °C, washed again, and incubated with TMB solution (Fisher Scientific) until color change was observed. The reaction was stopped with 5% phosphoric acid. Absorbance was measured at 450 nm using a Molecular Devices SpectraMax M3 microplate reader, and SoftMax Pro software was used to determine antibody titers.

### Parasitic burden

For in vivo studies, ears and draining lymph nodes were collected at the time of harvest (12 weeks post-infection for BALB/c mice). The tissues were homogenized using a cell strainer in 3 ml of Schneider’s Insect medium (Gibco, USA) supplemented with 20% heat-inactivated FBS and 1% penicillin/streptomycin. Each ear was separated into two sheets of dermis, washed in PBS containing 2% penicillin/streptomycin, and processed into small pieces. The tissue was then incubated in HBSS (Sigma-Aldrich, Cat: H9394) supplemented with 2 mM EDTA, 2% FBS, and 1% penicillin/streptomycin at 37 °C for 30 min, shaking at 250 rpm. After centrifugation, the cell pellet was incubated in DMEM + 2 mg/ml Collagenase A + 5% FBS + 1% penicillin/streptomycin at 37 °C for 1 hour, shaking at 250 rpm. The enzymatic reaction was stopped by adding FBS, and the tissue was mashed through a 70 μm strainer, then washed with DMEM + 20% FBS + 1% penicillin/streptomycin. The resulting lymph node or ear cell suspensions were serially diluted (1:20) in duplicates in two 96-well plates, ensuring that each sample was diluted across 24 wells. After 7 days of incubation at 26 °C, the plates were examined using an inverted microscope at 40x magnification. Parasite burden was determined from all six mice in the WT group, whereas four mice were available for the LmxTGT2-KO and AB groups due to loss of samples during processing of the mouse tissue to obtain axenic *L. mexicana* cells. The values reported in the graphs represent the highest logarithmic dilution showing viable parasites.

## Results

### The LmxTGT2 subunit is essential for the formation of Q-tRNAs in *L. mexicana*

We identified the LmxTGT1 (LmxM.29.1800) and LmxTGT2 (LmxM.27.0030) genes in the genome of *L. mexicana,* which show 67% and 42% identities to their orthologs from *T. brucei*, respectively. To study Q-tRNA modifications in *L. mexicana*, we focused on the LmxTGT2 subunit, as our previous studies in *T. brucei* demonstrated that both TbTGT1 and TbTGT2 subunits are essential for Q-tRNA formation [24]. We generated a gene knockout of the LmxTGT2 subunit in the promastigotes of *L. mexicana* using CRISPR/Cas9 (S1A Fig). The absence of the LmxTGT2 gene was confirmed by PCR analysis of genomic DNA (S1B Fig). Subsequently, an AB cell line was constructed using the pLEXSY vector containing a recoded Cas9-resistant copy of the LmxTGT2 gene flanked by the 5′ and 3′ untranslated regions (UTRs) of the A600 gene (LmxM.33.3640), which was previously reported to maintain stable expression throughout *Leishmania* differentiation [46].

To assess the effect of LmxTGT2 depletion, we monitored growth of the cultured procyclic promastigote stage, which showed no significant differences in growth rates among the WT, LmxTGT2 KO, and LmxTGT2 AB strains (Fig 1B). Next, we investigated the involvement of LmxTGT2 in the formation of the Q modification of tRNAs. Total RNA from WT, KO, and AB cell lines was analyzed using acrylamidophenyl boronic acid (APB)-affinity electrophoresis, followed by Northern blotting [38], which allows separation of the Q-modified tRNAs from their unmodified counterparts. The steady-state levels of Q-tRNA modifications in WT procyclic promastigotes varied among different tRNAs: Q-tRNA^Asp^, Q-tRNA^Asn^, and Q-tRNA^Tyr^ showed modification levels ranging from 42% to 66%, whereas Q-tRNA^His^ was close to the detection limit of the assay.

The complete absence of the bands corresponding to Q-modified tRNAs in the LmxTGT2 KO strain, along with a recovery of 44%–83% of WT levels (depending on the tRNA species) observed in the AB strain (Fig 1C), illustrates the direct role of LmxTGT2 in Q-tRNA formation.

### Q-tRNA levels increase during the differentiation from the promastigote to the amastigote stage

**Fig 2:**
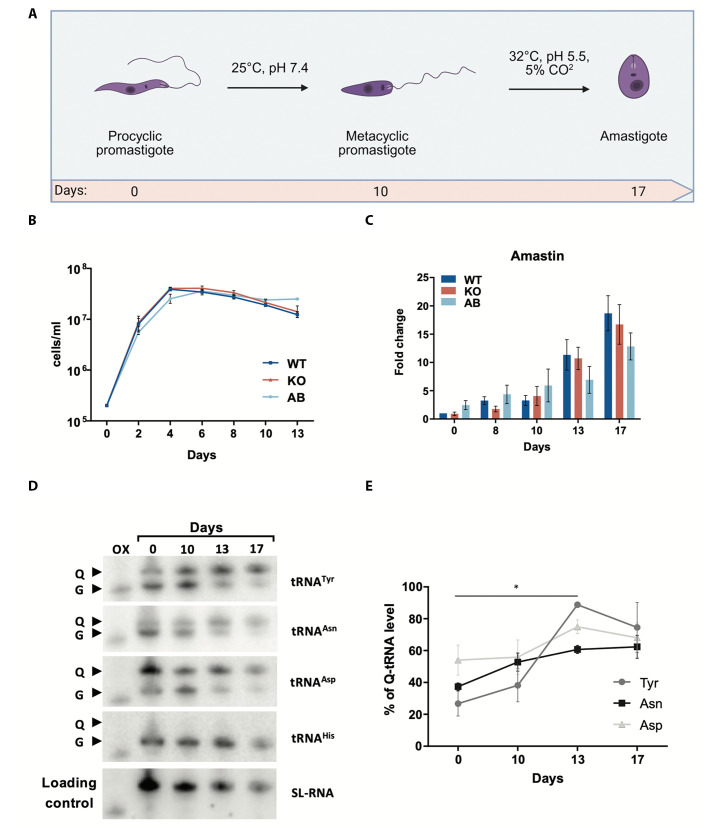
Q-tRNA levels increase during differentiation from the insect promastigote stage to the mammalian amastigote stage. **(A)** Schematic diagram of in vitro differentiation of *L. mexicana* (Created in BioRender. Paris, Z. (2026) https://BioRender.com/j85vrq5). **(B)** The growth curves of WT, LmxTGT2-KO, and AB cells exhibit no major alterations during in vitro differentiation into axenic metacyclic promastigotes. Procyclic promastigotes were maintained in the same cultivation medium until reaching the stationary phase, at which point they differentiated into metacyclic promastigotes (*n* = 3). **(C)** qPCR analysis of amastin marker expression during differentiation. Expression values were normalized to the WT level on day 0, which was set to 1. No significant differences between the tested cell lines were observed, indicating unaltered differentiation into axenic amastigotes (*n* = 5). **(D)** Northern blot analysis of total RNA from cells collected at 0, 10, 13, and 17 days of differentiation, separated on an APB gel. The oxidized RNA was used as a negative control (OX), and the splice leader (SL) RNA was used as a loading control. **(E)** Quantification of Q-tRNA levels from the northern blot experiments (as shown in D). Data were compared by two-way analysis of variance (ANOVA) with significant differences indicated (*P < 0.05, *n* = 3). The underlying data can be found in S1 Data.

To determine whether the levels of Q-tRNA modification change during the life cycle and whether its depletion affects the development of the parasite, we induced differentiation in the axenic culture. To simulate differentiation in vitro, promastigote cultures were left proliferating until they reach stationary phase and transformed into metacyclic promastigotes. Subsequently, cells were transferred into Schneider’s insect medium (pH 5.5) and incubated at 32 °C, which induced further differentiation into the axenic amastigote stage [39] (Fig 2A). However, the absence of Q-tRNA did not result in any noticeable changes in the growth or morphology of the LmxTGT2-KO parasites when growth was assessed daily up to day 13, within the same flask, and compared to the WT and AB strains (Fig 2B).

Although no obvious defects were observed during the differentiation in LmxTGT2-KO or AB, we validated the developmental progress by detecting stage-specific markers. As expected, expression of the amastigote-specific marker amastin increased during the differentiation into amastigotes. No significant changes in amastin expression were observed across the different cell lines (Fig 2C). Similarly, no cell line-specific differences were observed in the expression of the other stage-specific markers (S2 and S3 Figs). Collectively, these findings imply that although Q-tRNA modification increases during the life cycle, the absence of LmxTGT2 and subsequent lack of this modification does not substantially impact the differentiation process in vitro.

During the differentiation, we simultaneously examined variations in the Q-tRNA modification steady-state levels across different life stages of the WT. Using APB electrophoresis of the total RNA, we assessed the Q content of tRNA^Tyr^, tRNA^Asn^, tRNA^Asp^, and tRNA^His^ at various differentiation time points: day 0, day 10 (metacyclic promastigotes), day 13 (early amastigotes), and day 17 (amastigotes). The results revealed a global increase in the Q-tRNA modification levels throughout the life cycle, peaking at the amastigote stage. The increase was the most prominent in the tRNA^Tyr^, and less striking but still prominent in tRNA^Asp^ and tRNA^Asn^ (Fig 2D and 2E). Similar to the previous result (Fig 1C), Q modification in tRNA^His^ remained undetectable.

### LmxTGT2-KO compromises the ability of *L. mexicana* to infect macrophages in vitro

**Fig 3:**
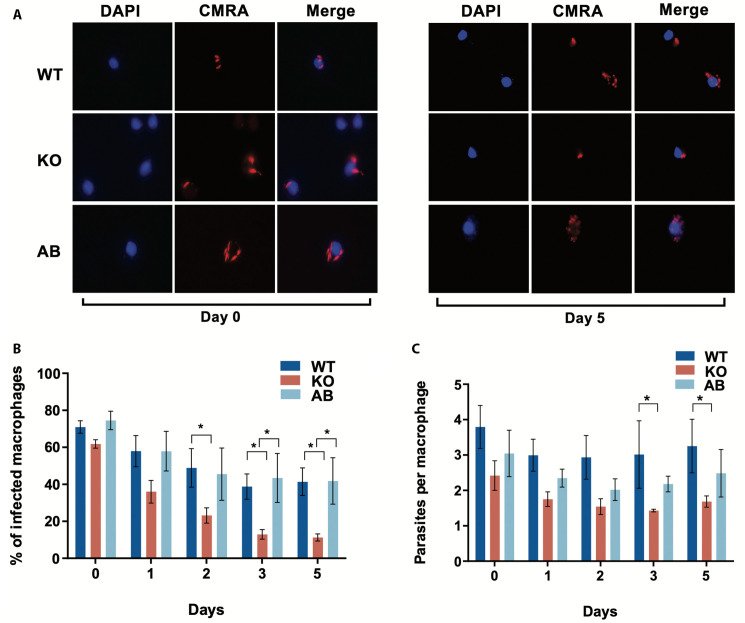
*L. mexicana* TGT2-KO has reduced infectivity and survival inside macrophages. **(A)** Fluorescence microscopy images of macrophages infected with WT, LmxTGT2-KO, and AB cells of *L. mexicana* are shown for illustration on day 0 and day 5 post-infection. Macrophage nuclei were stained with DAPI (blue), and parasites were labeled with CMRA orange cell tracker (red). **(B)** Quantification of macrophages infected with *L. mexicana*. Data represent three independent replicates of experimental infection assessed by fluorescence microscopy, showing the percentage of infected macrophages on days 0, 1, 2, 3, and 5 post-infections. A total of 100 macrophages were counted per sample. **(C)** The number of parasites per infected macrophage is shown. Data were compared by two-way analysis of variance (ANOVA), followed by Fisher’s LSD post hoc test, with significant differences indicated (*P < 0.05). The underlying data can be found in S1 Data.

Although the lack of Q-tRNAs did not affect cell proliferation in the axenic cell culture, the levels of Q-tRNAs changed during differentiation, suggesting a possible importance in the infectious stages of the parasite. Thus, we hypothesized that the absence of Q-tRNAs could be more relevant if the parasites were subjected to a challenging environment within their mammalian host cells. To test the potential impact of LmxTGT2-KO on the infectivity of the parasites, we conducted an in vitro infection of bone marrow-derived murine macrophages. The differentiated parasites were fluorescently labeled, and infection was monitored over five days following the initial infection. At the start of the experiment, 60%–70% of macrophages were infected (day 0) across all groups. However, the difference between the three tested groups became significant from day 2 to day 5 of infection (Fig 3A and 3B). Over time, the number of macrophages infected with LmxTGT2-KO parasites decreased substantially, resulting in only 11% infected macrophages on day 5, compared to 41% in the WT group (*P* = 0.0157) (Fig 3B). Correspondingly, the number of parasites detected inside a macrophage was comparable at the beginning of the infection but progressively decreased for LmxTGT2-KO parasites. WT parasites maintained higher intracellular parasite numbers, whereas the AB line displayed an intermediate phenotype and was not significantly different from either WT or KO at later time points (Fig 3C). Overall, these data indicate that LmxTGT2-KO parasites can initially infect macrophages at the same level as WT, but they are unable to survive inside the host cells for several days.

### Q-tRNA is important for cytosolic codon-biased translation

**Fig 4:**
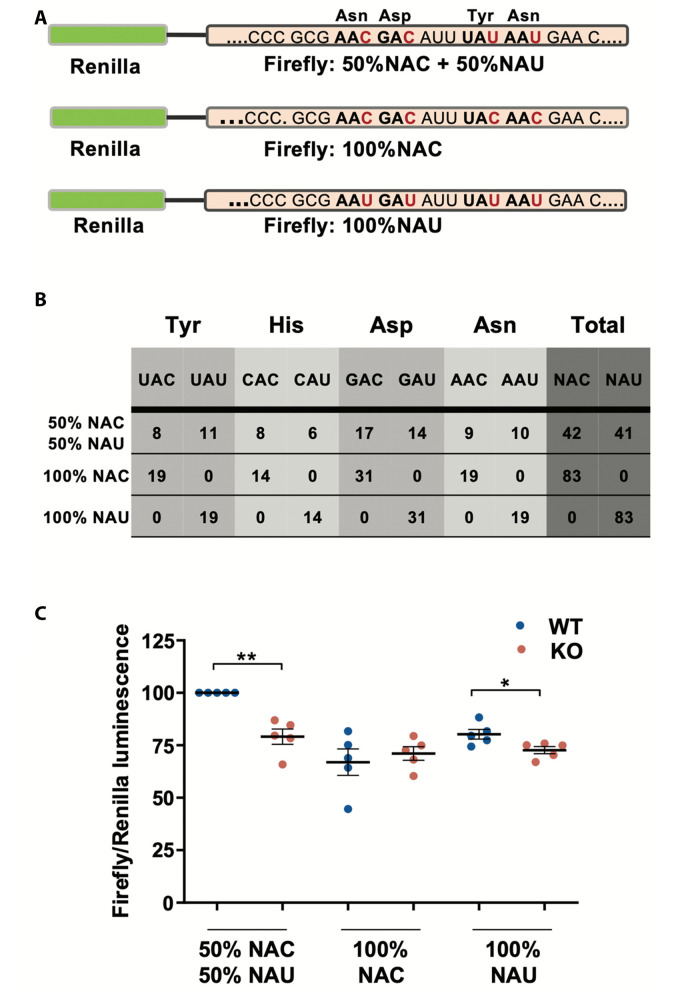
Q-tRNA is important for cytosolic codon-biased translation. **(A)** Schematic representation of a dual luciferase construct where Renilla luciferase (RLuc) is expressed in-frame with a codon-reengineered Firefly luciferase (FLuc). In the control construct, Q-dependent codons in the Fluc sequence are evenly distributed (50% NAC, 50% NAU), whereas in the test constructs, all Asn, Asp, Tyr, and His codons were recoded to contain exclusively either NAC or NAU. **(B)** Number of cognate codons for Q-tRNAs in the three test constructs. **(C)** Ratio of FLuc to RLuc luminescence measured in the cells, normalized to the control (50% NAC + 50% NAU), representing efficiency of translation of the reporter protein. Data were analyzed using Student *t* test, with significant differences indicated (**P < 0.01, *P < 0.05, *n* = 5). The underlying data can be found in S1 Data.

To investigate the role of Q in cytosolic codon-biased translation, we employed the dual luciferase reporter system, where the Renilla luciferase (RLuc) is fused to the Firefly luciferase (FLuc) with a linker (Fig 4A) [47]. Since these enzymes use different substrates, it is possible to quantify differential expression of these proteins simultaneously, in a luminescence assay. We generated codon re-engineered constructs, where the RLuc sequence was kept unaltered, but in the FLuc sequence, the cognate codons for all four Q-containing tRNAs (tRNA^His^_GUG_, tRNA^Asp^_GUC_, tRNA^Asn^_GUU_, tRNA^Tyr^_GUA_) were changed either to all NAC (100% NAC) or all NAU (100% NAU) codons, as opposed to the control 50% NAC + 50% NAU FLuc, which contained approximately equal number of NAC and NAU codons. The total numbers of NAC and NAU codons are enumerated in Fig 4B. We hypothesized, based on available literature and our previous results in *T. brucei*, that Q might be more efficient than G, in decoding of U-ending codons [24]. Thus, in the absence of Q-tRNAs we might observe a decreased translation of NAU luciferase, and subsequently, a decrease in the luminescence. We transfected these three constructs into WT and LmxTGT2-KO cells and measured FLuc luminescence, normalizing it to RLuc. The absence of LmxTGT2 resulted in an overall defect in translation, since luminescence dropped to 75% (*P* < 0.01) in KO cells with the control construct (50% NAC + 50% NAU). While the NAC construct did not show significant differences in signal between WT and LmxTGT2-KO cells, the NAU construct exhibited a significant decrease in Fluc luminescence in the absence of LmxTGT2 (Fig 4C). This suggests that NAU codons may be translated less efficiently without Q-tRNAs, as hypothesized above.

### The loss of Q‐tRNA modification alters proteome of *L. mexicana* during differentiation

**Fig 5:**
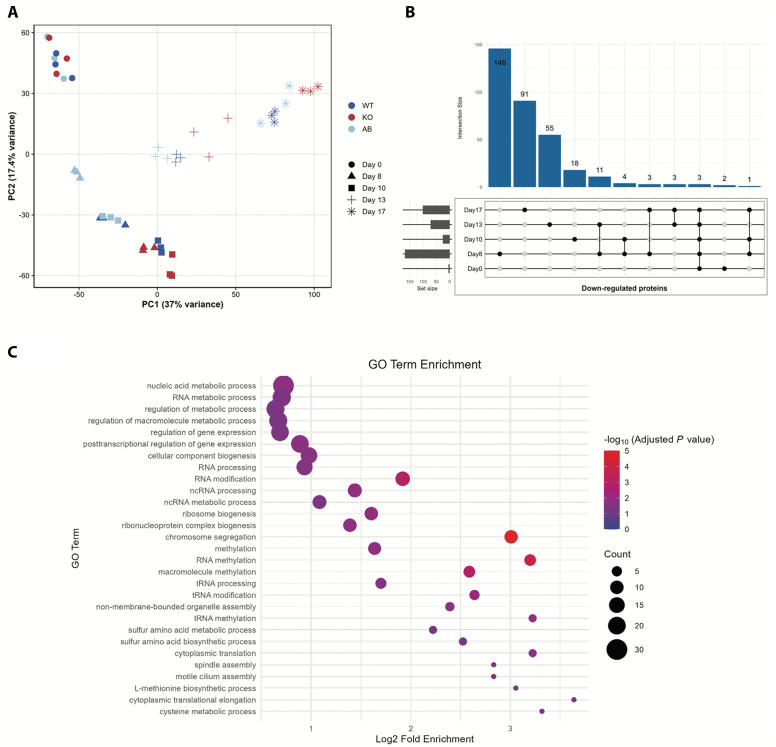
The absence of LmxTGT2 altered the protein profile during differentiation. (A) Principal component analysis (PCA) of WT, KO, and add-back (AB) cell lines across days 0, 8, 10, 13, and 17 of in vitro differentiation. PC1 and PC2 explain 37% and 17.4% of the total variance, respectively. PC1 reflects the major variance associated with differentiation, with samples progressing from left to right over time. Each point represents one biological replicate. PCA was performed in R using the prcomp function and visualized with ggplot2. **(B)** UpSet plot showing the number of downregulated proteins at different time points of differentiation in LmxTGT2-KO relative to WT and AB. Bars indicate the size of each intersection, and connected filled circles denote the time points that share common proteins. When no bar is present for a given combination, no common proteins were identified among those groups. Differential expression was defined as a fold change of ≥ 2 with statistical significance, as determined by Student *t* test (*P* < 0.05, *n* = 3). **(C)** Dot plot illustrating gene ontology (GO) enrichment analysis of proteins downregulated in LmxTGT2-KO cells throughout differentiation period (day 0 to day 17). GO-terms with less than two associated proteins were excluded, resulting in the removal of 22 terms. Of the 344 downregulated proteins, 313 were assigned to GO terms, while 31 remained uncharacterized. The size of the dots indicates the number of proteins linked to each GO term, and the color of the dots reflects the statistical significance of the enrichment. The underlying data can be found in S1 Data.

Since the luciferase assay indicated an effect of Q-tRNAs on codon-biased translation, we performed a proteomic analysis to investigate the consequences of Q-tRNA depletion on the proteome during the *L. mexicana* life cycle. This analysis had two main objectives: first, to identify differentially expressed proteins in LmxTGT2-KO relative to WT and AB at different time points; and second, to examine proteins that showed consistent changes throughout differentiation in LmxTGT2-KO (relative to day 0). Our results revealed significant differences in proteome composition, particularly on days 8, 13, and 17 of the differentiation process (Fig 5).

We performed PCA on the proteomics data collected across all time points (days 0, 8, 10, 13, and 17) to assess global proteomic changes during differentiation and evaluate the relationship between the WT, LmxTGT2-KO, and AB cell lines (Fig 5A). The first principal component (PC1), which accounted for 37% of the total variance, primarily reflected progression through in vitro differentiation. Samples from all cell lines shifted along PC1 over time. All three cell lines clustered tightly on day 0, indicating highly similar baseline proteomic profiles prior to differentiation. As differentiation progressed, WT and AB samples continued to cluster closely together, whereas KO samples diverged from this trajectory. Notably, the AB line consistently aligned with the WT line at later time points, demonstrating the restoration of a WT-like proteomic profile with the reintroduction of LmxTGT2. These data suggest that the observed proteomic differences in KO are differentiation-dependent and specifically attributable to the loss of LmxTGT2.

In terms of the overall proteome, we identified 7236 proteins. In KO relative to WT and AB, we observed 168 proteins downregulated on day 8, 72 proteins on day 13, and 101 proteins on day 17 (Fig 5B). In contrast, fewer proteins were upregulated, with a peak of 36 upregulated proteins on day 8, followed by 9 proteins on day 10, 4 proteins on day 13, and just 2 proteins on day 17 (2-fold change, *P* <0.05; S4 Fig). Notably, there were not many common proteins among different time points. Interestingly, only three proteins LmxTGT1, LmxTGT2, and an uncharacterized protein, were consistently downregulated across all time points, forming the only overlap between all groups (Fig 5B).

To assess proteomic alterations within LmxTGT2-KO during differentiation, we analyzed proteins that were consistently downregulated or upregulated relative to the LmxTGT2-KO starting point (day 0), while remaining unchanged in WT and AB. Between day 8 and day 17 of differentiation, a total of 344 proteins were significantly downregulated, while 131 proteins were significantly upregulated (2-fold change, *P <* 0.05).

To investigate the potential function of these downregulated proteins in LmxTGT2-KO cells, we performed the GO enrichment analysis. Overall, 313 proteins were mapped to GO terms, while 22 GO terms were excluded due to having only one associated protein. The most significantly enriched GO terms were related to RNA metabolism and processing, including RNA metabolic processes, RNA modification, ribosome biogenesis, and noncoding RNA processing, suggesting a disruption in RNA processing. Additionally, processes related to gene expression regulation, post-transcriptional control, and methylation were notably affected (Fig 5C).

**Fig 6:**
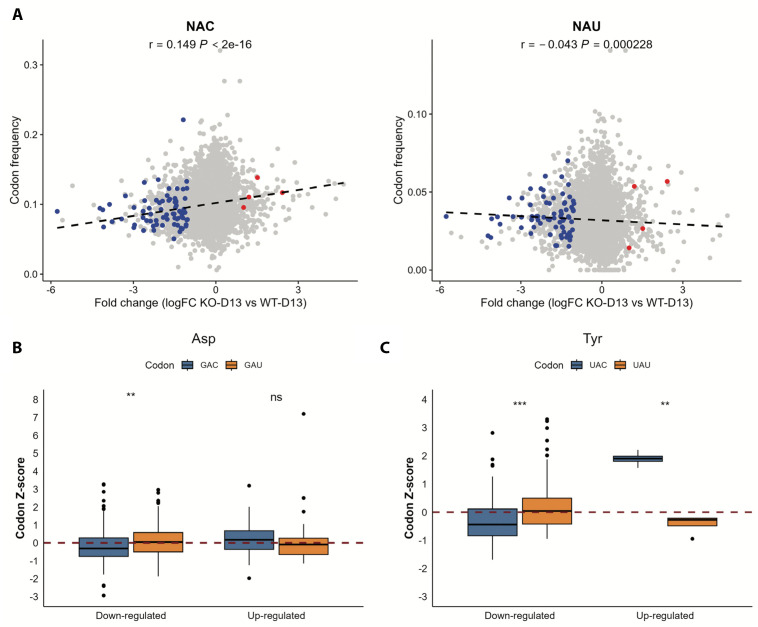
Q-tRNAs modulate translation during differentiation via codon bias. **(A)** Pearson correlation between codon frequency and protein fold change in upregulated and downregulated proteins for NAC and NAU codons in LmxTGT2-KO cells on day 13, relative to WT and AB. The Pearson correlation coefficient (r) with *P* value and linear regression line (black) are indicated. **(B)** Frequency of Asp codons on day 8 and Tyr codons on day 13 **(C)** was evaluated by calculating Z-scores for upregulated and downregulated proteins in KO cells. Here, the Z-score represents the degree to which observed codon usage deviates from the expected codon frequency in the genome. The center line indicates the median, the box shows the interquartile range (upper and lower quartiles), and individual dots represent outliers (minimum and maximum values). The data are based on three independent biological replicates used for proteomic analysis and were analyzed using Student *t* test. Significant differences are indicated (***P < 0.001, **P < 0.01, * *P* < 0.05, ns = not significant). The underlying data can be found in S1 Data.

To further investigate the impact of Q-tRNA on translation, we examined the correlation between changes in protein expression and the usage of Q-specific codons, focusing on NAC and NAU codons. We observed a trend suggesting a positive correlation between NAC codon frequency and protein levels on both day 8 (r = 0.166, *P* = 2 x 10^−16^) and day 13 (r = 0.149, *P* = 2 x 10^−16^). In contrast, NAU codons showed a trend towards negative correlation on day 8 (r = −0.005, *P* = 0.653) and day 13 (r = −0.043, *P* = 0.000228) (Figs 6A and S5A).

Since the effect of Q-modification may be specific to individual tRNAs and could be obscured in the broader analysis of NAC and NAU codons, we next focused on the specific codons involved. This analysis concentrated on amino acids decoded by Q-tRNAs, specifically tyrosine (Tyr), asparagine (Asn), aspartate (Asp), and histidine (His). Codon usage deviations were evaluated using the Z-score [48], which compares the observed frequency of specific codons with their expected frequency based on genome-wide codon usage. The analysis revealed significant differences in the usage of Asp codons in the proteome of day 8, since GAU codon is enriched in downregulated proteins (Fig 6B). Furthermore, Tyr codons showed pronounced enrichment of UAU in downregulated proteins, and enrichment of UAC in up-regulated proteins on day 13 (Fig 6C). Differences in codon usage at other time points were less pronounced (S5 and S6 Figs). In general, Q-tRNA-dependent U-ending codons were enriched in downregulated proteins, whereas C-ending codons were more prevalent in up-regulated proteins, in line with the original hypothesis.

### The ablation of LmxTGT2 impacts parasite infectivity and virulence in the mammalian host

**Fig 7:**
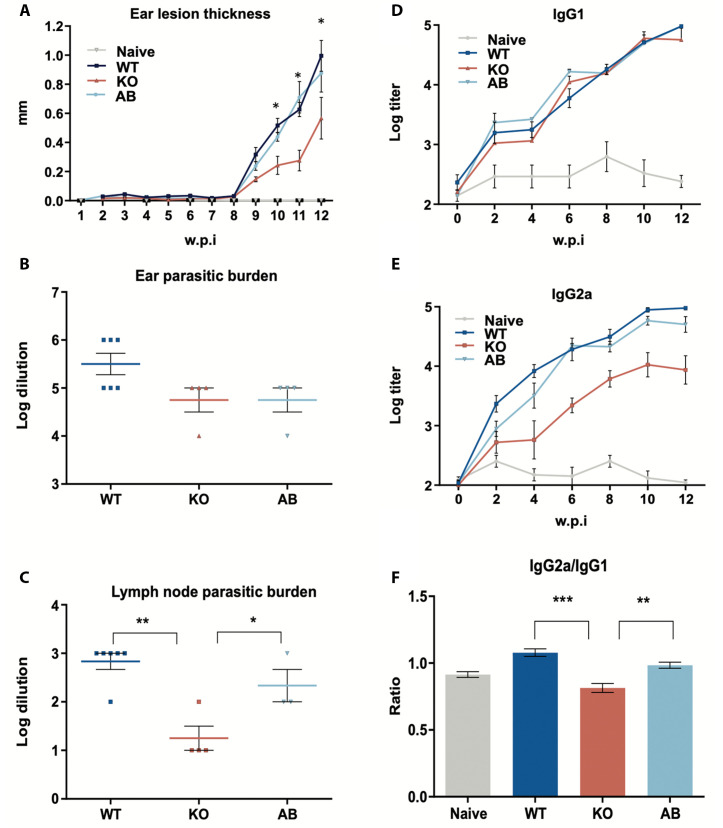
The ablation of LmxTGT2 impacts parasite infectivity and virulence in a mammalian host. **(A)** BALB/c mice were infected with *L. mexicana* wild-type (WT), add-back (AB), or LmxTGT2-KO parasites. Ear lesion size (in mm) was measured weekly over the course of infection (w.p.i. = weeks post-infection). Results shown are from one representative experiment out of two independent experiments (*n* = 6). **(B)** Parasite burden in ear tissue, assessed by serial dilution of lesion-derived material at week 12 following the end of the infection experiment (WT *n* = 6; KO *n* = 4; AB *n* = 4). **(C)** Parasite burden in the corresponding ear-draining lymph nodes (determined as in B). **(D)** Time course of the antibody response, showing IgG1 and **(E)** IgG2a levels measured by ELISA in sera from infected mice collected at two-week intervals (*n* = 4). **(F)** IgG2a/IgG1 ratio was calculated from the data shown in panels D and E. Data were analyzed using Student *t* test, and significance is indicated (***P < 0.001, ** *P* < 0.01, *P < 0.05). The underlying data can be found in S1 Data.

To determine whether the decreased infection observed in macrophages could be replicated in vivo and prompted by the profound effects on the LmxTGT2-KO proteome, an infection study was performed in BALB/c mice to investigate a role of LmxTGT2 in *L. mexicana* virulence. Parasites were administered intradermally into an ear of each mouse. Lesions became apparent after 8 weeks in all groups and continued to increase in size. From the ninth week onward, notable variations in lesion size were observed, with mice infected with LmxTGT2-KO parasites developing lesions approximately half the size of those caused by WT and AB strains. This difference became statistically significant from week 10 onward (Fig 7A).

The assessment of parasite load through a serial dilution assay did not reveal significant changes in the number of parasites in the ear (Fig 7B), despite the larger lesions observed in the WT and AB groups compared to LmxTGT2-KO. Interestingly, a lower number of parasites was detected in the lymph nodes in LmxTGT2-KO compared to WT and AB (Fig 7C). To evaluate the inflammatory response in infected mice, we performed an enzyme-linked immunosorbent assay (ELISA) for IgG1 and IgG2a in the serum. Throughout the infection period, IgG1 levels steadily increased in all tested groups (Fig 7D). In contrast, IgG2a titers were significantly lower in LmxTGT2-KO animals compared to WT and AB mice (Fig 7E). The ratio of IgG2a to IgG1 was calculated to be significantly reduced in mice infected with the LmxTGT2-KO strain (Fig 7F). This ratio is an important indicator of the immune response profile, where a higher IgG2a/IgG1 ratio typically reflects a Th1-biased (protective) immune response, whereas a lower ratio indicates a Th2 (anti-inflammatory) response [49,50].

## Discussion

The wide range of biological phenomena associated with Q has made it challenging to pinpoint a single, unified role for this modification across species [17]. However, a dynamic pattern of Q-tRNA modification has been observed in diverse organisms, suggesting a broader regulatory role across eukaryotes and linking environmental cues and developmental programs to the fine-tuning of translation.

In this work, we show that in the protozoan parasite *L. mexicana*, Q-tRNA levels gradually increased during differentiation, reaching their highest levels in the axenic amastigote stage. This finding suggests that Q-tRNAs play a role in controlling stage-specific development. Indeed, deletion of the noncatalytically active subunit of TGT in *L. mexicana* (LmxTGT2) abolished Q-tRNA formation but showed no apparent growth defects (Figs 1 and 2), suggesting that Q-modification may not be essential for basal proliferation and differentiation in vitro, but may be required under changing environment or nutrient-limiting conditions. This interpretation is consistent with findings in other systems, where deletion of TGT subunits (QTRT1 or QTRT2) did not impair proliferation under normal conditions [30,51].

Q-tRNAs correspond to single tRNA isoacceptors decoding Asn, Asp, Tyr, or His codons; all found in two-codon boxes (NAU and NAC). The N7-deaza guanosine-derived analog Q replaces guanosine at position 34 (wobble base), subtly altering base-pairing dynamics. While Q in most organisms does not alter the Watson–Crick face of the anticodon, it enhances wobble pairing with U over C, favoring decoding of U-ending codons in most contexts [17]. However, in *S. pombe*, the opposite pattern has been reported, where Q preferentially enhances the translation of C-ending codons [33]. As shown here, we observed a significant reduction in NAU-driven Firefly luciferase activity in Q-tRNA deficient *L. mexicana* cells (Fig 4), analogous to our previous observations in *T. brucei* [24]. However, even the NAC-only construct exhibited reduced translation regardless of the presence or absence of Q-tRNAs, suggesting that codon optimality itself contributes to translational output independent of Q-modification. Interestingly, reporter constructs with a balanced 50:50 ratio of NAU and NAC codons also exhibited a reduction of translation in LmxTGT2-KO cells, indicating a general defect on translation in the absence of Q-tRNAs.

One plausible mechanism underlying this broader translational defect is that loss of Q increases the susceptibility of cognate tRNAs to ribonuclease-mediated cleavage, resulting in the accumulation of tRNA-derived fragments that can inhibit translation initiation and impair global protein synthesis [52,53]. In addition, Q deficiency or reduction of its glycosylated forms has been associated with altered elongation dynamics and reduced translational efficiency at the proteome level [29,51]. Together, these observations indicate that Q-tRNA modification contributes to overall translational efficiency and is not limited to codon-biased decoding.

In agreement with the dual reporter assay, our proteomic analysis revealed that proteins downregulated in Q-tRNA deficient cells were significantly enriched for NAU codons, whereas proteins upregulated under these conditions showed enrichment for NAC codons (Figs 6, S5, and S6). Although codon usage alone does not fully explain the observed protein expression levels, these trends are consistent with the idea that Q-tRNA modification promotes efficient translation of NAU-rich transcripts. The effect becomes more pronounced when individual codon pairs are considered, suggesting that the effect of Q-modification varies depending on the specific tRNA involved.

To further interpret the observed proteomic changes, we next asked whether these differences corresponded to particular biological processes. GO-term analysis revealed that proteins associated with RNA metabolism and RNA processing were preferentially affected (Fig 5C), suggesting that Q-tRNA depletion impacts pathways that rely on tightly regulated protein synthesis. Many of these proteins frequently function within large, multi-component ribonucleoprotein complexes whose proper assembly depends on coordinated production of their constituent subunits. Recent work has demonstrated that many such complexes assemble co-translationally and are particularly sensitive to perturbations in translational dynamics or efficiency [54]. In this context, even modest defects in translational robustness caused by Q-tRNA depletion could preferentially destabilize RNA-processing pathways that rely on precise stoichiometry and timing of protein production. Consistent with this model, studies of tRNA modification loss have shown that altered decoding dynamics can induce proteotoxic stress and compromise proteome integrity, disproportionately affecting complex cellular functions [55,56].

**Fig 8:**
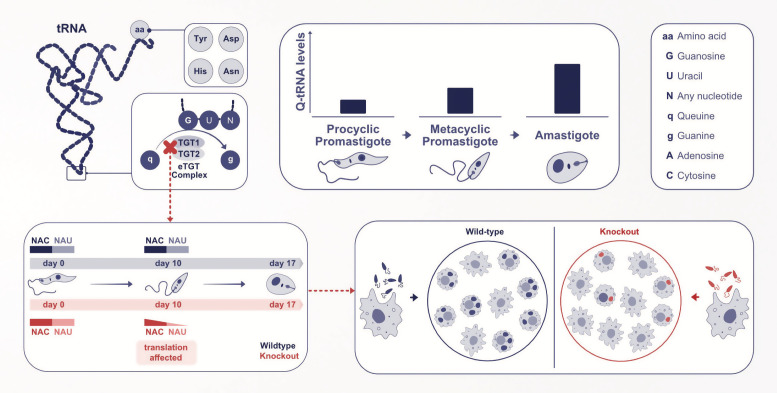
Model illustrating the role of queuosine (Q) tRNA modification in the virulence of *L. mexicana.* Q-tRNA levels increase significantly during the differentiation from the procyclic promastigotes (insect stage) to the axenic amastigotes (mammalian stage), underscoring the importance of Q-tRNA modification for parasite adaptation and infectivity. Loss of Q modification via LmxTGT2 knockout disrupts codon-biased translational control, leading to substantial changes in the proteome. Specifically, downregulated proteins were enriched in NAU codons decoded by Q-tRNA, while upregulated proteins predominantly contained NAC codons. The resulting codon-specific imbalance impairs the parasite’s ability to establish infection and maintain virulence. These findings demonstrate that Q modification functions as a novel post-transcriptional regulatory mechanism that fine-tunes translation during *Leishmania* life cycle, compensating for the lack of classical transcriptional control and promoting adaptation to the host environment.

As defined by Grosjean and Westhof [57], codons with A and/or U at the first two positions are thermodynamically “weak” whereas those with G and/or C are “strong”. In this context, Asn (AAU/AAC) and Tyr (UAU/UAC) codons are weak; Asp (GAU/GAC) and His (CAU/CAC) are intermediate. It has been proposed that Q-modified tRNAs may help buffer such stability differences, enhancing decoding kinetics across codon families with varying strength [57]. While our study does not directly address this mechanism, the preferential reduction in translation of NAU-rich transcripts observed in Q-tRNA deficient cells is consistent with the idea that Q-tRNAs enhance decoding of weaker codons, fine-tuning decoding efficiency, which is particularly important for codons that are thermodynamically less favorable. Together, these data support a model where Q-tRNA modification facilitates codon-specific translation during developmental transition between the insect and mammalian stage (Fig 8). Its absence compromises the efficient decoding of specific codons under a changing environment, while remaining dispensable during homeostatic growth. Future work combining ribosome profiling with enhanced protein annotation will be required to resolve how Q-tRNA influences translation in a codon context and transcript-specific manner.

An open question is how Q-tRNA modification levels increase during *Leishmania* differentiation, given that under normal conditions tRNAs are not fully modified. One possible explanation is that the TGT complex itself becomes upregulated during differentiation; however, our proteomic data does not support this. Considering our previous observations in the *T. brucei* system [58], more likely scenarios may be either increased dwell time of tRNAs in the nucleus or changes in nutrient levels when cells develop into metacyclics and then into amastigotes, which are characterized by slower growth and reduced metabolic rates and higher sensitivity to perturbations [59]. Notably, in the LmxTGT2 knockout, we observed a partial reduction in the overall levels of a subpopulation of tRNAs that typically undergo Q modification, consistent with previous findings that Q contributes to tRNA stability [52]. This supports the interpretation that Q-tRNA depletion primarily exerts a subtle, codon-specific effect on translation, with broader translational impairments arising as a secondary consequence.

Given the proposed role of Q-tRNA modification in oxidative stress responses [60] and the exposure of *Leishmania* parasites to environmental changes following macrophage infection, we explored the effects of oxidative stress on reporter expression. However, oxidative stress markedly reduced expression of both luciferase reporters, preventing reliable assessment of codon-dependent effects. More broadly, stress responses in trypanosomatids are associated with extensive translational remodeling and repression [61,62], which may mask subtle codon-specific effects in the current dual-reporter system.

Several studies suggest that Q plays a role in modulating virulence across both prokaryotic and eukaryotic pathogens [31,60,63,64]. To our knowledge, the only eukaryotic pathogen that has been investigated in the context of Q is *E. histolytica*, where queuine supplementation slightly increased Q-tRNA levels, enhanced oxidative stress responses, and counterintuitively reduced virulence by downregulating key virulence factors [60]. In our report, the most pronounced phenotypic effect observed was the reduced infectivity of LmxTGT2-KO parasites. However, none of the known virulence factors were among the most affected proteins, preventing a straightforward explanation for the observed decrease in infectivity. Notably, a substantial proportion of the significantly affected genes encode hypothetical proteins or proteins of unknown function, which leaves open the possibility that they may play important roles in *Leishmania*-specific differentiation or infection.

Interestingly, the LmxTGT2-KO parasites caused significantly smaller ear lesions in infected mice compared with mice infected with WT or AB strains (Fig 7A). However, this reduction in lesion size occurred despite comparable parasite burdens, suggesting that Q-tRNA deficiency reduces the parasite’s capacity to induce lesion-associated pathology, potentially through an altered host inflammatory response. Although Q-tRNA-depleted parasites exhibited reduced survival and lower infectivity in macrophages in vitro (Fig 3), this phenotype was not reflected in the parasite burdens observed in vivo. These findings indicate that lesion size was influenced not only by parasite fitness but also by differences in host inflammatory responses. Since the outcome of *Leishmania* infection is largely determined by the host–parasite interaction, the severity of the disease can vary. Therefore, the larger lesions observed in control infections may result from an exacerbated immune response rather than a higher parasite load [65–67]. Indeed, the various strains in this study showed comparable levels of IgG1, but IgG2a levels were significantly lower in LmxTGT2-KO-infected mice. As a result, the IgG2a/IgG1 ratio, a marker of pro-inflammatory, IFN-γ-mediated Th1 responses [49,50], was significantly reduced (Fig 7F), suggesting a weakened immune activation in the absence of Q-tRNAs. While this may explain the significantly smaller lesions observed in mice infected with the KO parasites, the underlying immune mechanisms are likely more complex, and a detailed characterization will be required in future studies.

Although our data support a primary role for Q-tRNA modification in codon-biased translation and infectivity, they do not preclude the possibility that queuosine (Q) may have additional, yet unidentified biological functions. Increasing evidence shows that RNA modifications once considered tRNA-specific can also occur in other RNA classes; for example, m⁶A is now known to play broad roles in mRNA stability and translation [68]. Early work in *Shigella flexneri* even speculated that Q might be present on the *virF* mRNA, even though this was never experimentally confirmed [63,69]. While intriguing, determining whether Q or Q-related derivatives occur in mRNAs remains speculative and is out of the scope of this study.

In conclusion, our study provides the first direct evidence that queuosine tRNA modification regulates infectivity in the eukaryotic pathogen *L. mexicana* through codon-biased translational control. Q-tRNA levels increase significantly during differentiation from the insect into the mammalian-infective stage, and loss of Q modification via LmxTGT2 knockout impairs protein synthesis, leading to a codon-specific imbalance in the proteome that affects parasite virulence (Fig 8). These findings establish Q as an additional, novel layer of gene expression regulation in different life-cycle stages of *Leishmania*, providing new insight into how these parasites fine-tune translation to adapt to host environments in the absence of classical transcriptional control.

## Supporting information

S1 Fig Design and confirmation of LmxTGT2 knockout in *L. mexicana.* **(A)** Schematic illustration of the strategy used to generate and validate LmxTGT2 knockout (KO) cells. Positions of PCR primers used for genotyping are indicated (Created in BioRender. Paris, Z. (2026) https://BioRender.com/ceb39l3). **(B)** PCR-based amplification followed by DNA electrophoresis reveals the presence or absence of specific genomic sequences in wild-type (WT) and knockout (KO) cells. Product sizes (in base pairs) are indicated to confirm the specificity of the knockout strategy.
(TIFF)

S2 Fig Proteomic analysis of stage-specific marker expression during differentiation. **(A–C)** Proteomic expression levels of stage-specific markers Amastin, PfrD1, and Sherp during *L. mexicana* differentiation. The underlying data can be found in S1 Data.
(TIFF)

S3 Fig Depletion of Q-tRNAs does not affect stage-specific marker expression. **(A)** qPCR analysis of PfrD1 and Sherp, markers of the promastigote and metacyclic stages, during *L. mexicana* differentiation. The underlying data can be found in S1 Data.
(TIFF)

S4 Fig Overlap of up-regulated proteins during in vitro differentiation in LmxTGT2-KO parasites. UpSet plot showing the number of up-regulated proteins at different time points of differentiation in LmxTGT2-KO relative to WT and AB. Bars indicate the size of each intersection, and connected filled circles denote the time points that share common proteins. When no bar is present for a given combination, no common proteins were identified among those groups. Differential expression was defined as a fold change of ≥2 with statistical significance, as determined by Student *t* test (*P* < 0.05, *n* = 3). The underlying data can be found in S1 Data.
(TIFF)

S5 Fig Regulation of translation via codon bias in the absence of LmxTGT2 (day 8). **(A)** Pearson correlation between NAC and NAU codon frequency and protein abundance in LmxTGT2-KO cells on day 8, relative to WT and AB. The Pearson correlation coefficient (r) with *P* value and linear regression line (black) are indicated. **(B–D)** Codon frequency of Tyr, His, and Asn shown as Z-scores, in upregulated and downregulated proteins in LmxTGT2-KO cells on day 8, relative to wild-type (WT) and add-back (AB). **(E)** Codon frequency of all NAC and NAU codons, shown as Z-scores, in upregulated and downregulated proteins on day 8. The center line indicates the median, the box shows the interquartile range (upper and lower quartiles), and individual dots represent outliers (minimum and maximum values). The data are based on three independent biological replicates used for proteomic analysis and were analyzed using Student *t* test. Significant differences are indicated as follows: (**P < 0.01, * *P* < 0.05, ns = not significant). The underlying data can be found in S1 Data.
(TIFF)

S6 Fig Regulation of translation via codon bias in the absence of LmxTGT2 (day 13). **(A–C)** Codon frequency of Asp, His, and Asn codons, shown as Z-scores, in upregulated and downregulated proteins in LmxTGT2-KO cells on day 13, relative to wild-type (WT) and add-back (AB). **(D)** Codon frequency of all NAC and NAU codons, shown as Z-scores, in upregulated and downregulated proteins in LmxTGT2-KO cells on day 13, relative to wild-type (WT) and add-back (AB). The center line indicates the median, the box shows the interquartile range (upper and lower quartiles), and individual dots represent outliers (minimum and maximum values). The data are based on three independent biological replicates used for proteomic analysis and were analyzed using Student *t* test. Significant differences are indicated as follows: (**P < 0.01, * *P* < 0.05, ns = not significant). The underlying data can be found in S1 Data.
(TIFF)

S1 Table List of oligonucleotides used for cell line generation, PCR validation, and Northern blotting. (TIFF)

S1 Data Raw measurements underlying the quantitative analyses presented in Figs 1B, 1C, 2B–2E, 3B, 3C, 4C, 5A–5C, 6A–6C, 7A–7F, S2A–S2C, S3A, S4, S5A–S5E, S6A–S6D. (XLSX)

S1 Raw Images Uncropped, unprocessed images corresponding to Figs 1C, 2D, and S1B. (PDF)

## Abbreviations

AB: add-back
APB: aminophenylboronic acid
DIA: data-independent acquisition
FBS: fetal bovine serum
Fluc: Firefly luciferase
GO: Gene Ontology
KO: knock-out
LC-MS/MS: liquid chromatography–tandem mass spectrometry
PCA: principal component analysis
qPCR: quantitative reverse transcription PCR
QNG1: queuine-nucleoside hydrolase
Rluc: Renilla luciferase
sgRNA: single guide RNA
SL-RNA: splice leader RNA
TGT: tRNA-guanine transglycosylase
UTRs: untranslated regions
WT: wild-type.

## References

[1] Pace D. Leishmaniasis. J Infect. 2014;69 Suppl 1:S10-8. doi: 10.1016/j.jinf.2014.07.016 25238669

[2] Kevric I, Cappel MA, Keeling JH. New world and old world *Leishmania* infections: a practical review. Dermatol Clin. 2015;33(3):579–93. doi: 10.1016/j.det.2015.03.018 26143433

[3] Torres-Guerrero E, Quintanilla-Cedillo MR, Ruiz-Esmenjaud J, Arenas R. *Leishmaniasis*: a review. F1000Res. 2017;6:750. doi: 10.12688/f1000research.11120.1 28649370 PMC5464238

[4] Ponte-Sucre A, Gamarro F, Dujardin J-C, Barrett MP, López-Vélez R, García-Hernández R, et al. Drug resistance and treatment failure in leishmaniasis: a 21st century challenge. PLoS Negl Trop Dis. 2017;11(12):e0006052. doi: 10.1371/journal.pntd.0006052 29240765 PMC5730103

[5] Ayala A, Llanes A, Lleonart R, Restrepo CM. Advances in *Leishmania* vaccines: current development and future prospects. Pathogens. 2024;13(9):812. doi: 10.3390/pathogens13090812 39339003 PMC11435054

[6] Wolf Nassif P, DE Mello TFP, Navasconi TR, Mota CA, Demarchi IG, Aristides SMA, et al. Safety and efficacy of current alternatives in the topical treatment of cutaneous leishmaniasis: a systematic review. Parasitology. 2017;144(8):995–1004. doi: 10.1017/S0031182017000385 28367792

[7] Silva-Moreira AL, Serravite AM, Rios-Barros LV, de Menezes JPB, Horta MF, Castro-Gomes T. New insights into the life cycle, host cell tropism, and infection amplification of *Leishmania* spp. Infect Immun. 2025;93(7):e0012325. doi: 10.1128/iai.00123-25 40512038 PMC12234434

[8] De Pablos LM, Ferreira TR, Walrad PB. Developmental differentiation in *Leishmania* lifecycle progression: post-transcriptional control conducts the orchestra. Curr Opin Microbiol. 2016;34:82–9. doi: 10.1016/j.mib.2016.08.004 27565628

[9] Kramer S. Developmental regulation of gene expression in the absence of transcriptional control: the case of kinetoplastids. Mol Biochem Parasitol. 2012;181(2):61–72. doi: 10.1016/j.molbiopara.2011.10.002 22019385

[10] Maslov DA, Opperdoes FR, Kostygov AY, Hashimi H, Lukeš J, Yurchenko V. Recent advances in trypanosomatid research: genome organization, expression, metabolism, taxonomy and evolution. Parasitology. 2019;146(1):1–27. doi: 10.1017/S0031182018000951 29898792

[11] David M, Gabdank I, Ben-David M, Zilka A, Orr I, Barash D, et al. Preferential translation of Hsp83 in Leishmania requires a thermosensitive polypyrimidine-rich element in the 3’ UTR and involves scanning of the 5’ UTR. RNA. 2010;16(2):364–74. doi: 10.1261/rna.1874710 20040590 PMC2811665

[12] Zilka A, Garlapati S, Dahan E, Yaolsky V, Shapira M. Developmental regulation of heat shock protein 83 in *Leishmania*. 3’ processing and mRNA stability control transcript abundance, and translation id directed by a determinant in the 3’-untranslated region. J Biol Chem. 2001;276(51):47922–9. doi: 10.1074/jbc.M108271200 11598129

[13] Trenaman A, Tinti M, Wall RJ, Horn D. Post-transcriptional reprogramming by thousands of mRNA untranslated regions in trypanosomes. Nat Commun. 2024;15(1):8113. doi: 10.1038/s41467-024-52432-0 39285175 PMC11405848

[14] Gustilo EM, Vendeix FA, Agris PF. tRNA’s modifications bring order to gene expression. Curr Opin Microbiol. 2008;11(2):134–40. doi: 10.1016/j.mib.2008.02.003 18378185 PMC2408636

[15] Fergus C, Barnes D, Alqasem MA, Kelly VP. The queuine micronutrient: charting a course from microbe to man. Nutrients. 2015;7(4):2897–929. doi: 10.3390/nu7042897 25884661 PMC4425180

[16] Rashad S. Queuosine tRNA modification: connecting the microbiome to the translatome. BioEssays. 2024;47(2). doi: 10.1002/bies.202400213 PMC11755703 39600051

[17] Ehrenhofer-Murray AE. Queuine: a bacterial nucleobase shaping translation in eukaryotes. J Mol Biol. 2025;437(16):168985. doi: 10.1016/j.jmb.2025.168985 39956693

[18] Hung S-H, Elliott GI, Ramkumar TR, Burtnyak L, McGrenaghan CJ, Alkuzweny S, et al. Structural basis of Qng1-mediated salvage of the micronutrient queuine from queuosine-5’-monophosphate as the biological substrate. Nucleic Acids Res. 2023;51(2):935–51. doi: 10.1093/nar/gkac1231 36610787 PMC9881137

[19] Chen Y-C, Kelly VP, Stachura SV, Garcia GA. Characterization of the human tRNA-guanine transglycosylase: confirmation of the heterodimeric subunit structure. RNA. 2010;16(5):958–68. doi: 10.1261/rna.1997610 20354154 PMC2856889

[20] Stengl B, Meyer EA, Heine A, Brenk R, Diederich F, Klebe G. Crystal structures of tRNA-guanine transglycosylase (TGT) in complex with novel and potent inhibitors unravel pronounced induced-fit adaptations and suggest dimer formation upon substrate binding. J Mol Biol. 2007;370(3):492–511. doi: 10.1016/j.jmb.2007.04.008 17524419

[21] Johannsson S, Neumann P, Ficner R. Crystal Structure of the human tRNA guanine transglycosylase catalytic subunit QTRT1. Biomolecules. 2018;8(3):81. doi: 10.3390/biom8030081 30149595 PMC6165067

[22] Sievers K, Welp L, Urlaub H, Ficner R. Structural and functional insights into human tRNA guanine transgylcosylase. RNA Biol. 2021;18(sup1):382–96. doi: 10.1080/15476286.2021.1950980 34241577 PMC8677009

[23] Kaczmarczyk I, Koziej Ł, Glatt S. It takes two to tRNAgo. Structure. 2024;32(3):260–2. doi: 10.1016/j.str.2024.02.005 38458158

[24] Kulkarni S, Rubio MAT, Hegedűsová E, Ross RL, Limbach PA, Alfonzo JD, et al. Preferential import of queuosine-modified tRNAs into *Trypanosoma brucei* mitochondrion is critical for organellar protein synthesis. Nucleic Acids Res. 2021;49(14):8247–60. doi: 10.1093/nar/gkab567 34244755 PMC8373054

[25] Dirheimer G, Baranowski W, Keith G. Variations in tRNA modifications, particularly of their queuine content in higher eukaryotes. Its relation to malignancy grading. Biochimie. 1995;77(1–2):99–103. doi: 10.1016/0300-9084(96)88111-9 7599283

[26] Langgut W, Reisser T, Nishimura S, Kersten H. Modulation of mammalian cell proliferation by a modified tRNA base of bacterial origin. FEBS Lett. 1993;336(1):137–42. doi: 10.1016/0014-5793(93)81627-c 8262197

[27] Morris RC, Brown KG, Elliott MS. The effect of queuosine on tRNA structure and function. J Biomol Struct Dyn. 1999;16(4):757–74. doi: 10.1080/07391102.1999.10508291 10217448

[28] Dixit S, Kessler AC, Henderson J, Pan X, Zhao R, D’Almeida GS, et al. Dynamic queuosine changes in tRNA couple nutrient levels to codon choice in *Trypanosoma brucei*. Nucleic Acids Res. 2021;49(22):12986–99. doi: 10.1093/nar/gkab1204 34883512 PMC8682783

[29] Tuorto F, Legrand C, Cirzi C, Federico G, Liebers R, Müller M, et al. Queuosine-modified tRNAs confer nutritional control of protein translation. EMBO J. 2018;37(18):e99777. doi: 10.15252/embj.201899777 30093495 PMC6138434

[30] Suzuki T, Yashiro Y, Kikuchi I, Ishigami Y, Saito H, Matsuzawa I, et al. Complete chemical structures of human mitochondrial tRNAs. Nat Commun. 2020;11(1):4269. doi: 10.1038/s41467-020-18068-6 32859890 PMC7455718

[31] Díaz-Rullo J, González-Pastor JE. tRNA queuosine modification is involved in biofilm formation and virulence in bacteria. Nucleic Acids Res. 2023;51(18):9821–37. doi: 10.1093/nar/gkad667 37638766 PMC10570037

[32] Zaborske JM, DuMont VLB, Wallace EWJ, Pan T, Aquadro CF, Drummond DA. A nutrient-driven tRNA modification alters translational fidelity and genome-wide protein coding across an animal genus. PLoS Biol. 2014;12(12):e1002015. doi: 10.1371/journal.pbio.1002015 25489848 PMC4260829

[33] Müller M, Legrand C, Tuorto F, Kelly VP, Atlasi Y, Lyko F, et al. Queuine links translational control in eukaryotes to a micronutrient from bacteria. Nucleic Acids Res. 2019;47(7):3711–27. doi: 10.1093/nar/gkz063 30715423 PMC6468285

[34] Cirzi C, Dyckow J, Legrand C, Schott J, Guo W, Perez Hernandez D, et al. Queuosine-tRNA promotes sex-dependent learning and memory formation by maintaining codon-biased translation elongation speed. EMBO J. 2023;42(19):e112507. doi: 10.15252/embj.2022112507 37609797 PMC10548180

[35] Ishemgulova A, Hlaváčová J, Majerová K, Butenko A, Lukeš J, Votýpka J, et al. CRISPR/Cas9 in *Leishmania mexicana*: a case study of LmxBTN1. PLoS One. 2018;13(2):e0192723. doi: 10.1371/journal.pone.0192723 29438445 PMC5811015

[36] Peng D, Tarleton R. EuPaGDT: a web tool tailored to design CRISPR guide RNAs for eukaryotic pathogens. Microbial Genomics. 2015;1(4):null. 10.1099/mgen.0.000033 PMC5320623 28348817

[37] Chomczynski P, Sacchi N. Single-step method of RNA isolation by acid guanidinium thiocyanate-phenol-chloroform extraction. Anal Biochem. 1987;162(1):156–9. doi: 10.1006/abio.1987.9999 2440339

[38] Igloi GL, Kössel H. Affinity electrophoresis for monitoring terminal phosphorylation and the presence of queuosine in RNA: application of polyacrylamide containing a covalently bound boronic acid. Nucleic Acids Res. 1985;13(19):6881–98. doi: 10.1093/nar/13.19.6881 2414733 PMC322011

[39] Bates PA. Complete developmental cycle of *Leishmania mexicana* in axenic culture. Parasitology. 1994;108 (Pt 1):1–9. doi: 10.1017/s0031182000078458 8152848

[40] Zhang X, Goncalves R, Mosser DM. The isolation and characterization of murine macrophages. Curr Protoc Immunol. 2008;Chapter 14:14.1.1-14.1.14. doi: 10.1002/0471142735.im1401s83 19016445 PMC2834554

[41] Dagley MJ, Saunders EC, Simpson KJ, McConville MJ. High-content assay for measuring intracellular growth of *Leishmania* in human macrophages. Assay Drug Dev Technol. 2015;13(7):389–401. doi: 10.1089/adt.2015.652 26247370

[42] Wiśniewski JR, Ostasiewicz P, Mann M. High recovery FASP applied to the proteomic analysis of microdissected formalin fixed paraffin embedded cancer tissues retrieves known colon cancer markers. J Proteome Res. 2011;10(7):3040–9. doi: 10.1021/pr200019m 21526778

[43] Demichev V, Messner CB, Vernardis SI, Lilley KS, Ralser M. DIA-NN: neural networks and interference correction enable deep proteome coverage in high throughput. Nat Methods. 2020;17(1):41–4. doi: 10.1038/s41592-019-0638-x 31768060 PMC6949130

[44] Perez-Riverol Y, Bai J, Bandla C, García-Seisdedos D, Hewapathirana S, Kamatchinathan S, et al. The PRIDE database resources in 2022: a hub for mass spectrometry-based proteomics evidences. Nucleic Acids Res. 2022;50(D1):D543–52. doi: 10.1093/nar/gkab1038 34723319 PMC8728295

[45] Alexaki A, Kames J, Holcomb DD, Athey J, Santana-Quintero LV, Lam PVN, et al. Codon and Codon-Pair usage tables (CoCoPUTs): facilitating genetic variation analyses and recombinant gene design. J Mol Biol. 2019;431(13):2434–41. doi: 10.1016/j.jmb.2019.04.021 31029701

[46] Murray A, Fu C, Habibi G, McMaster WR. Regions in the 3’ untranslated region confer stage-specific expression to the *Leishmania mexicana* a600-4 gene. Mol Biochem Parasitol. 2007;153(2):125–32. doi: 10.1016/j.molbiopara.2007.02.010 17433460

[47] Sherf BA, Leuther KK, Niles RK, Roesler W. Dual-Luciferase reporter assay: an advanced co-reporter technology integrating Firefly and Renilla luciferase assays. Promega Notes. 1996;57:2–8.

[48] Small-Saunders JL, Sinha A, Bloxham TS, Hagenah LM, Sun G, Preiser PR, et al. tRNA modification reprogramming contributes to artemisinin resistance in *Plasmodium falciparum*. Nat Microbiol. 2024;9(6):1483–98. doi: 10.1038/s41564-024-01664-3 38632343 PMC11153160

[49] Volpedo G, Pacheco-Fernandez T, Holcomb EA, Zhang W-W, Lypaczewski P, Cox B, et al. Centrin-deficient Leishmania mexicana confers protection against New World cutaneous leishmaniasis. NPJ Vaccines. 2022;7(1):32. doi: 10.1038/s41541-022-00449-1 35236861 PMC8891280

[50] Rostamian M, Sohrabi S, Kavosifard H, Niknam HM. Lower levels of IgG1 in comparison with IgG2a are associated with protective immunity against *Leishmania tropica* infection in BALB/c mice. J Microbiol Immunol Infect. 2017;50(2):160–6. doi: 10.1016/j.jmii.2015.05.007 26066544

[51] Zhao X, Ma D, Ishiguro K, Saito H, Akichika S, Matsuzawa I, et al. Glycosylated queuosines in tRNAs optimize translational rate and post-embryonic growth. Cell. 2023;186(25):5517-5535.e24. doi: 10.1016/j.cell.2023.10.026 37992713

[52] Wang X, Matuszek Z, Huang Y, Parisien M, Dai Q, Clark W, et al. Queuosine modification protects cognate tRNAs against ribonuclease cleavage. RNA. 2018;24(10):1305–13. doi: 10.1261/rna.067033.118 29970597 PMC6140461

[53] Ivanov P, Emara MM, Villen J, Gygi SP, Anderson P. Angiogenin-induced tRNA fragments inhibit translation initiation. Mol Cell. 2011;43(4):613–23. doi: 10.1016/j.molcel.2011.06.022 21855800 PMC3160621

[54] Shiber A, Döring K, Friedrich U, Klann K, Merker D, Zedan M, et al. Cotranslational assembly of protein complexes in eukaryotes revealed by ribosome profiling. Nature. 2018;561(7722):268–72. doi: 10.1038/s41586-018-0462-y 30158700 PMC6372068

[55] Nedialkova DD, Leidel SA. Optimization of Codon Translation rates via tRNA modifications maintains proteome integrity. Cell. 2015;161(7):1606–18. doi: 10.1016/j.cell.2015.05.022 26052047 PMC4503807

[56] Chou H-J, Donnard E, Gustafsson HT, Garber M, Rando OJ. Transcriptome-wide analysis of roles for tRNA modifications in translational regulation. Mol Cell. 2017;68(5):978-992.e4. doi: 10.1016/j.molcel.2017.11.002 29198561 PMC5728682

[57] Grosjean H, Westhof E. An integrated, structure- and energy-based view of the genetic code. Nucleic Acids Res. 2016;44(17):8020–40. doi: 10.1093/nar/gkw608 27448410 PMC5041475

[58] Hegedűsová E, Kulkarni S, Burgman B, Alfonzo JD, Paris Z. The general mRNA exporters Mex67 and Mtr2 play distinct roles in nuclear export of tRNAs in *Trypanosoma brucei*. Nucleic Acids Research. 2019;47(16):8620–31. doi: 10.1093/nar/gkz671 31392978 PMC6794378

[59] Saunders EC, Ng WW, Kloehn J, Chambers JM, Ng M, McConville MJ. Induction of a stringent metabolic response in intracellular stages of *Leishmania mexicana* leads to increased dependence on mitochondrial metabolism. PLoS Pathog. 2014;10(1):e1003888. doi: 10.1371/journal.ppat.1003888 24465208 PMC3900632

[60] Nagaraja S, Cai MW, Sun J, Varet H, Sarid L, Trebicz-Geffen M, et al. Queuine is a nutritional regulator of *Entamoeba histolytica* response to oxidative stress and a virulence attenuator. mBio. 2021;12(2):e03549-20. doi: 10.1128/mBio.03549-20 33688012 PMC8092309

[61] Kramer S, Queiroz R, Ellis L, Webb H, Hoheisel JD, Clayton C, et al. Heat shock causes a decrease in polysomes and the appearance of stress granules in trypanosomes independently of eIF2(alpha) phosphorylation at Thr169. J Cell Sci. 2008;121(Pt 18):3002–14. doi: 10.1242/jcs.031823 18713834 PMC2871294

[62] Cloutier S, Laverdière M, Chou M-N, Boilard N, Chow C, Papadopoulou B. Translational control through eIF2alpha phosphorylation during the *Leishmania* differentiation process. PLoS One. 2012;7(5):e35085. doi: 10.1371/journal.pone.0035085 22693545 PMC3365078

[63] Durand JM, Dagberg B, Uhlin BE, Björk GR. Transfer RNA modification, temperature and DNA superhelicity have a common target in the regulatory network of the virulence of *Shigella flexneri*: the expression of the virF gene. Mol Microbiol. 2000;35(4):924–35. doi: 10.1046/j.1365-2958.2000.01767.x 10692168

[64] Hurt JK, Olgen S, Garcia GA. Site-specific modification of *Shigella flexneri* virF mRNA by tRNA-guanine transglycosylase in vitro. Nucleic Acids Res. 2007;35(14):4905–13. doi: 10.1093/nar/gkm473 17626052 PMC1950534

[65] Weinkopff T, Konradt C, Christian DA, Discher DE, Hunter CA, Scott P. *Leishmania* major infection-induced VEGF-A/VEGFR-2 signaling promotes lymphangiogenesis that controls disease. J Immunol. 2016;197(5):1823–31. doi: 10.4049/jimmunol.1600717 27474074 PMC5001553

[66] Baldwin TM, Elso C, Curtis J, Buckingham L, Handman E. The site of *Leishmania* major infection determines disease severity and immune responses. Infect Immun. 2003;71(12):6830–4. doi: 10.1128/IAI.71.12.6830-6834.2003 14638769 PMC308923

[67] Alcoforado Diniz J, Chaves MM, Vaselek S, Miserani Magalhães RD, Ricci-Azevedo R, de Carvalho RVH, et al. Protein methyltransferase 7 deficiency in *Leishmania* major increases neutrophil associated pathology in murine model. PLoS Negl Trop Dis. 2021;15(3):e0009230. doi: 10.1371/journal.pntd.0009230 33651805 PMC7954300

[68] Roundtree IA, Evans ME, Pan T, He C. Dynamic RNA modifications in gene expression regulation. Cell. 2017;169(7):1187–200. doi: 10.1016/j.cell.2017.05.045 28622506 PMC5657247

[69] Durand JM, Okada N, Tobe T, Watarai M, Fukuda I, Suzuki T, et al. vacC, a virulence-associated chromosomal locus of *Shigella flexneri*, is homologous to tgt, a gene encoding tRNA-guanine transglycosylase (Tgt) of *Escherichia coli* K-12. J Bacteriol. 1994;176(15):4627–34. doi: 10.1128/jb.176.15.4627-4634.1994 8045893 PMC196283

