# Supplementary figures and images for: Queuosine tRNA modification regulates translational adaptation and virulence of Leishmania mexicana
Source: PLoS Biol. 2026 Jul 17;24(7):e3003887. doi: 10.1371/journal.pbio.3003887 (PMC13421765; doi:10.1371/journal.pbio.3003887)

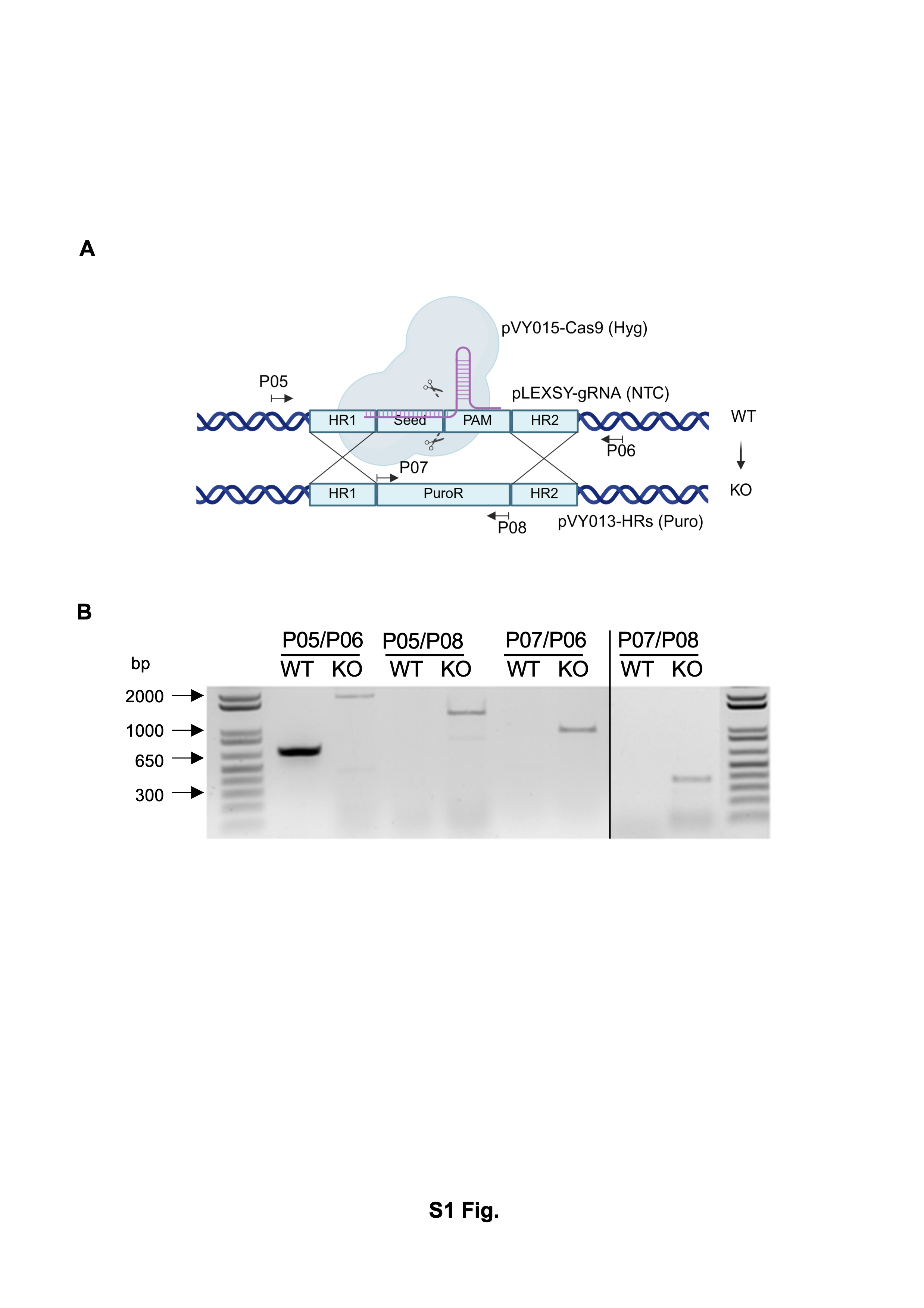

Supplement: S1 Fig — (A) Schematic illustration of the strategy used to generate and validate LmxTGT2 knockout (KO) cells. Positions of PCR primers used for genotyping are indicated (Created in BioRender. Paris, Z. (2026) https://BioRender.com/ceb39l3). (B) PCR-based amplification followed by DNA electrophoresis reveals the presence or absence of specific genomic sequences in wild-type (WT) and knockout (KO) cells. Product sizes (in base pairs) are indicated to confirm the specificity of the knockout strategy. (TIFF) [file pbio.3003887.s001.tiff]

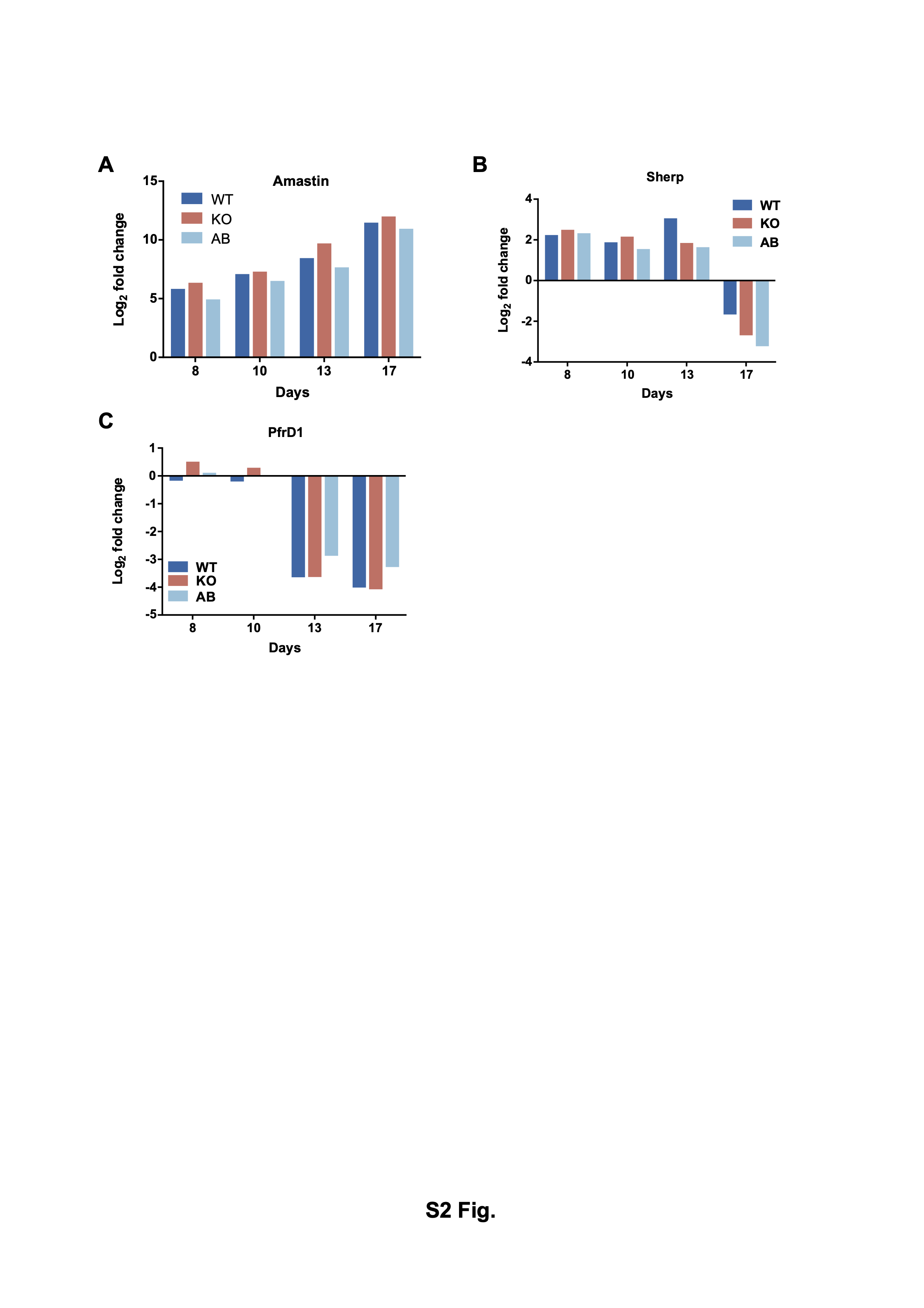

Supplement: S2 Fig — (A–C) Proteomic expression levels of stage-specific markers Amastin, PfrD1, and Sherp during L. mexicana differentiation. The underlying data can be found in S1 Data. (TIFF) [file pbio.3003887.s002.tiff]

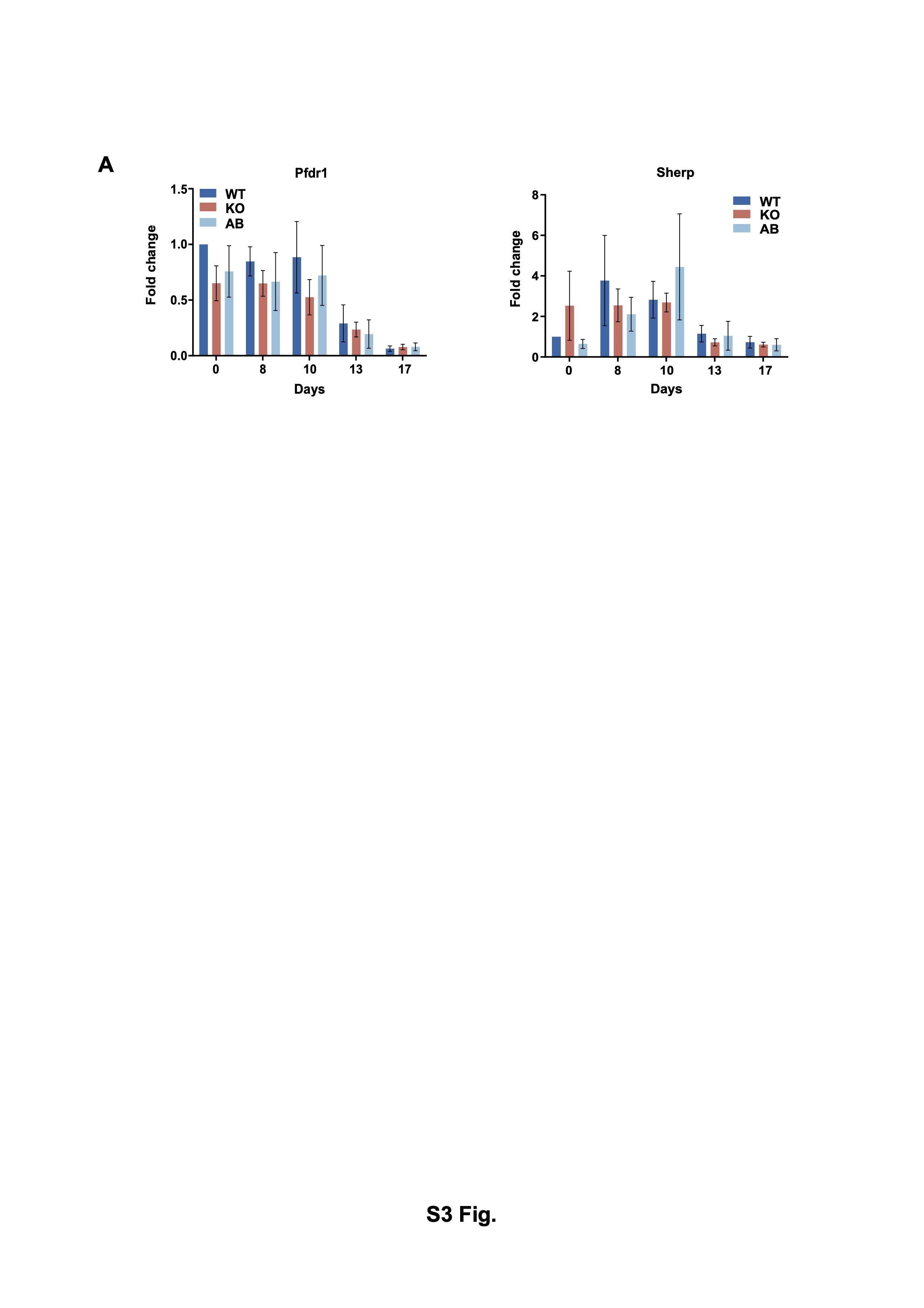

Supplement: S3 Fig — (A) qPCR analysis of PfrD1 and Sherp, markers of the promastigote and metacyclic stages, during L. mexicana differentiation. The underlying data can be found in S1 Data. (TIFF) [file pbio.3003887.s003.tiff]

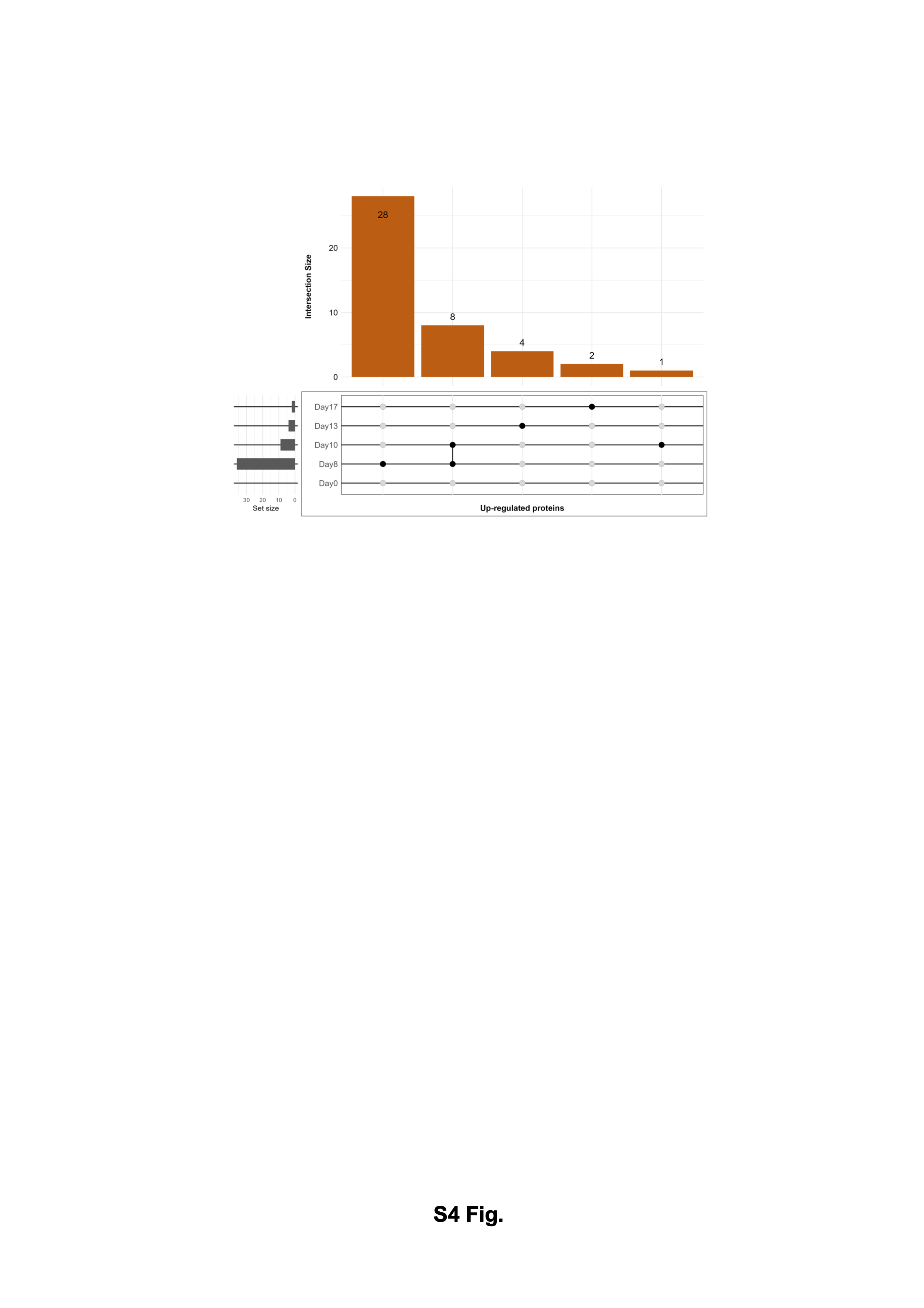

Supplement: S4 Fig — UpSet plot showing the number of up-regulated proteins at different time points of differentiation in LmxTGT2-KO relative to WT and AB. Bars indicate the size of each intersection, and connected filled circles denote the time points that share common proteins. When no bar is present for a given combination, no common proteins were identified among those groups. Differential expression was defined as a fold change of ≥2 with statistical significance, as determined by Student t test (P < 0.05, n = 3). The underlying data can be found in S1 Data. (TIFF) [file pbio.3003887.s004.tiff]

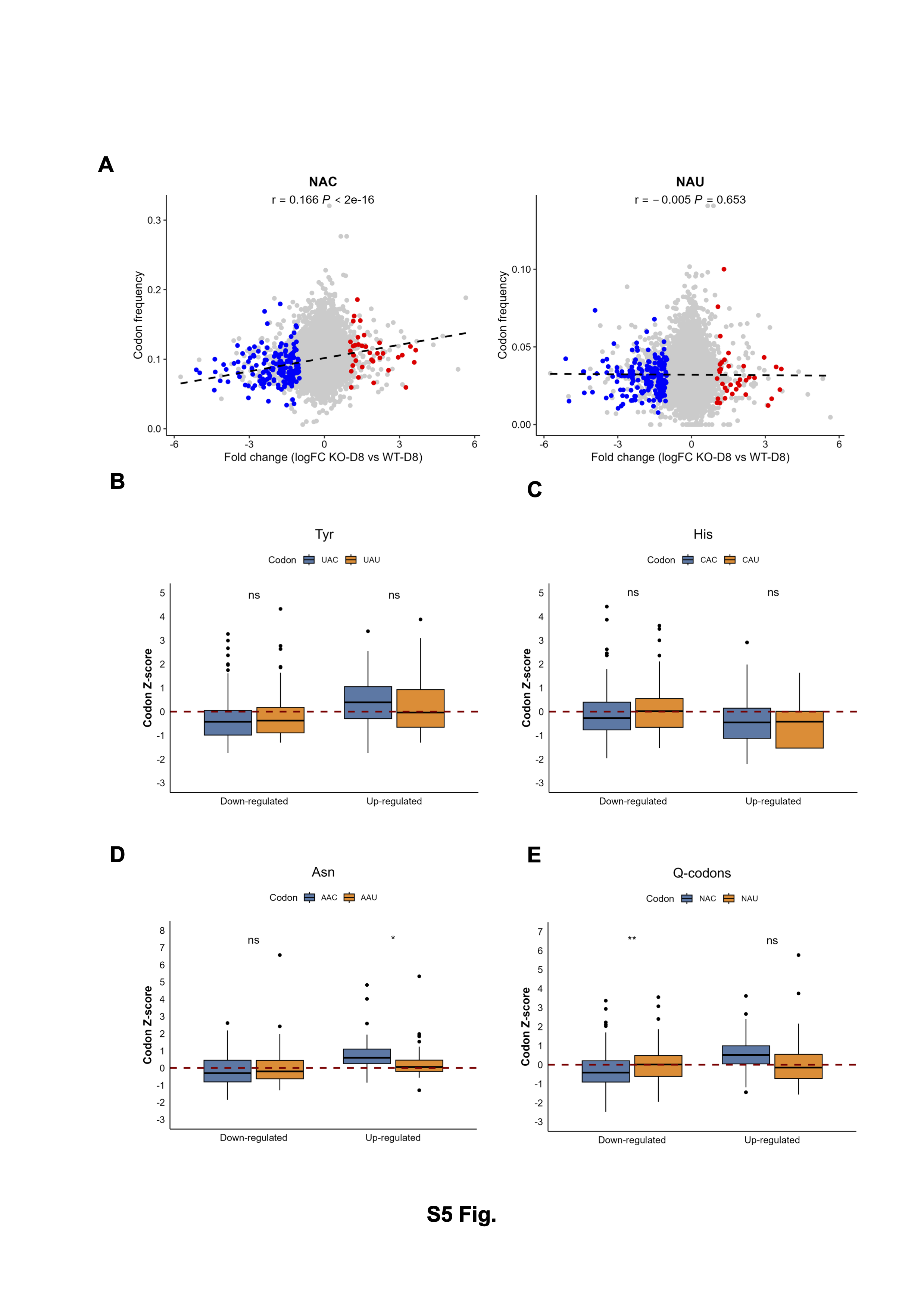

Supplement: S5 Fig — (A) Pearson correlation between NAC and NAU codon frequency and protein abundance in LmxTGT2-KO cells on day 8, relative to WT and AB. The Pearson correlation coefficient (r) with P value and linear regression line (black) are indicated. (B–D) Codon frequency of Tyr, His, and Asn shown as Z-scores, in upregulated and downregulated proteins in LmxTGT2-KO cells on day 8, relative to wild-type (WT) and add-back (AB). (E) Codon frequency of all NAC and NAU codons, shown as Z-scores, in upregulated and downregulated proteins on day 8. The center line indicates the median, the box shows the interquartile range (upper and lower quartiles), and individual dots represent outliers (minimum and maximum values). The data are based on three independent biological replicates used for proteomic analysis and were analyzed using Student t test. Significant differences are indicated as follows: (**P < 0.01, * P < 0.05, ns = not significant). The underlying data can be found in S1 Data. (TIFF) [file pbio.3003887.s005.tiff]

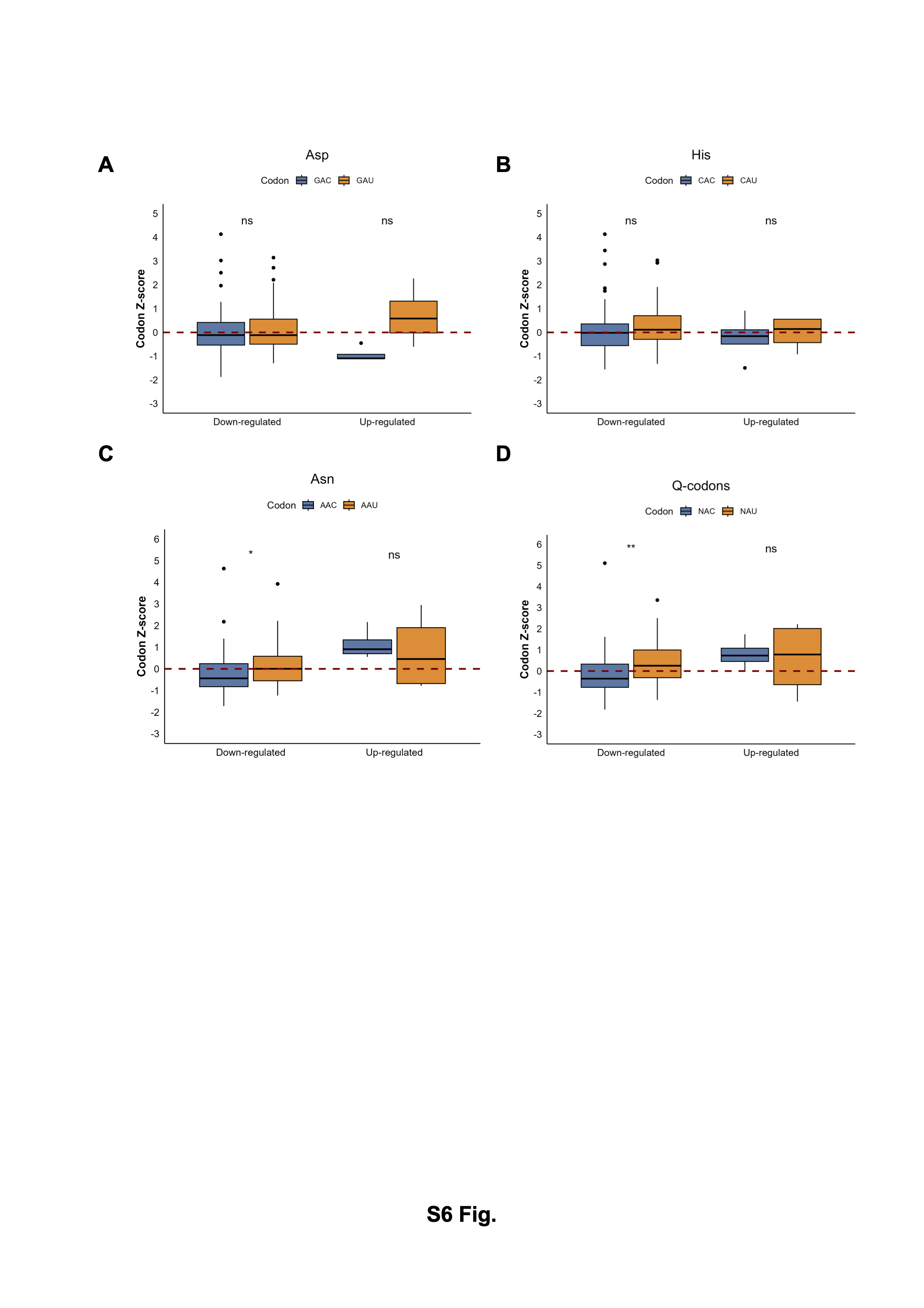

Supplement: S6 Fig — (A–C) Codon frequency of Asp, His, and Asn codons, shown as Z-scores, in upregulated and downregulated proteins in LmxTGT2-KO cells on day 13, relative to wild-type (WT) and add-back (AB). (D) Codon frequency of all NAC and NAU codons, shown as Z-scores, in upregulated and downregulated proteins in LmxTGT2-KO cells on day 13, relative to wild-type (WT) and add-back (AB). The center line indicates the median, the box shows the interquartile range (upper and lower quartiles), and individual dots represent outliers (minimum and maximum values). The data are based on three independent biological replicates used for proteomic analysis and were analyzed using Student t test. Significant differences are indicated as follows: (**P < 0.01, * P < 0.05, ns = not significant). The underlying data can be found in S1 Data. (TIFF) [file pbio.3003887.s006.tiff]

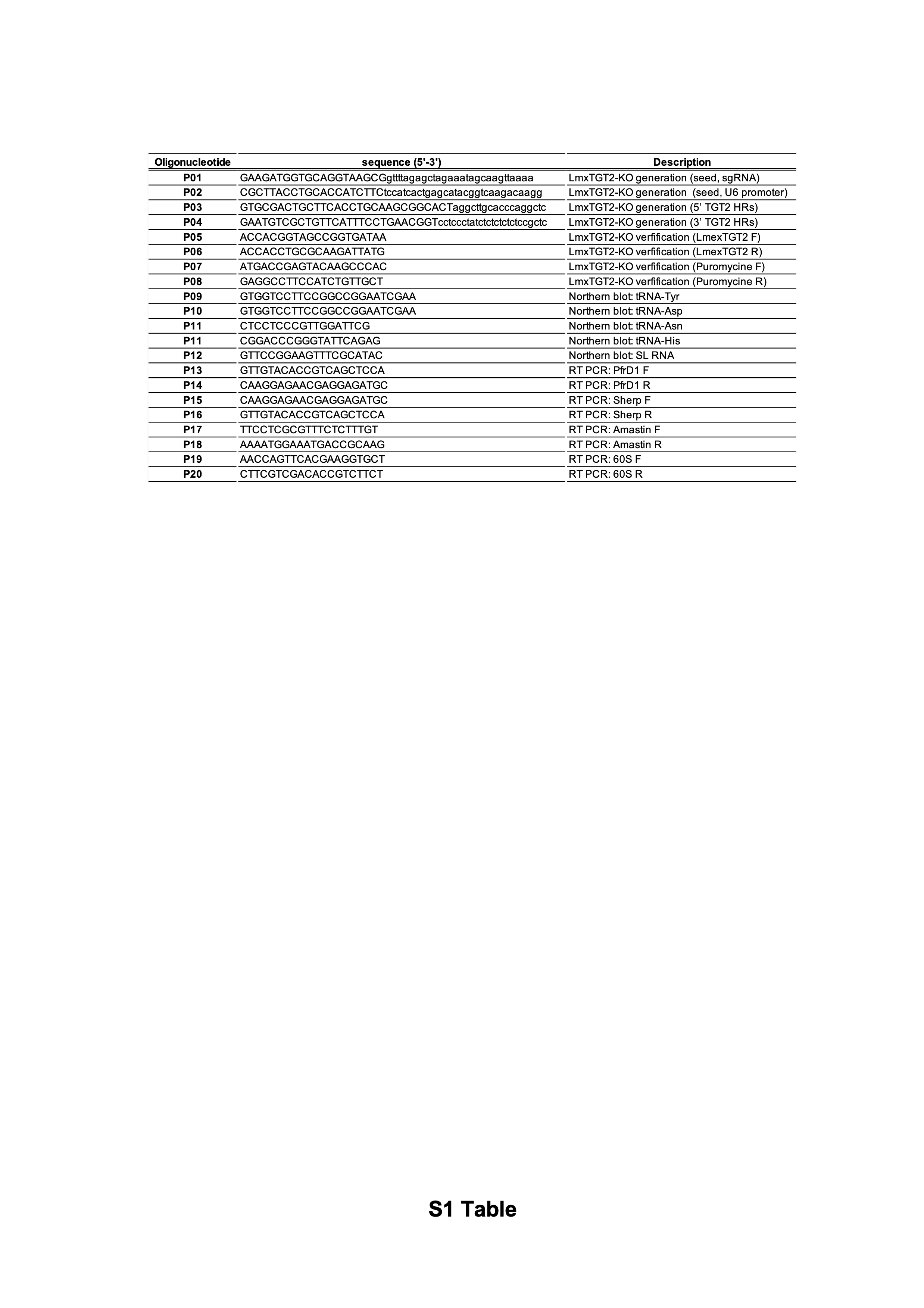

Supplement: S1 Table — (TIFF) [file pbio.3003887.s007.tiff]

Fig. 1C Northern blot

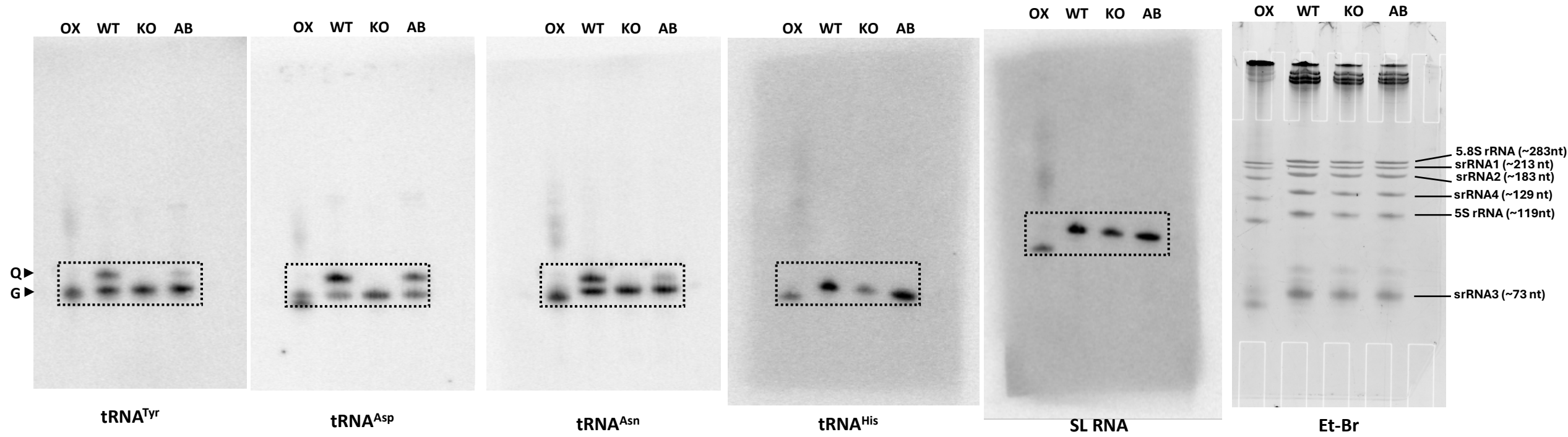

Fig. 2D Northern blot

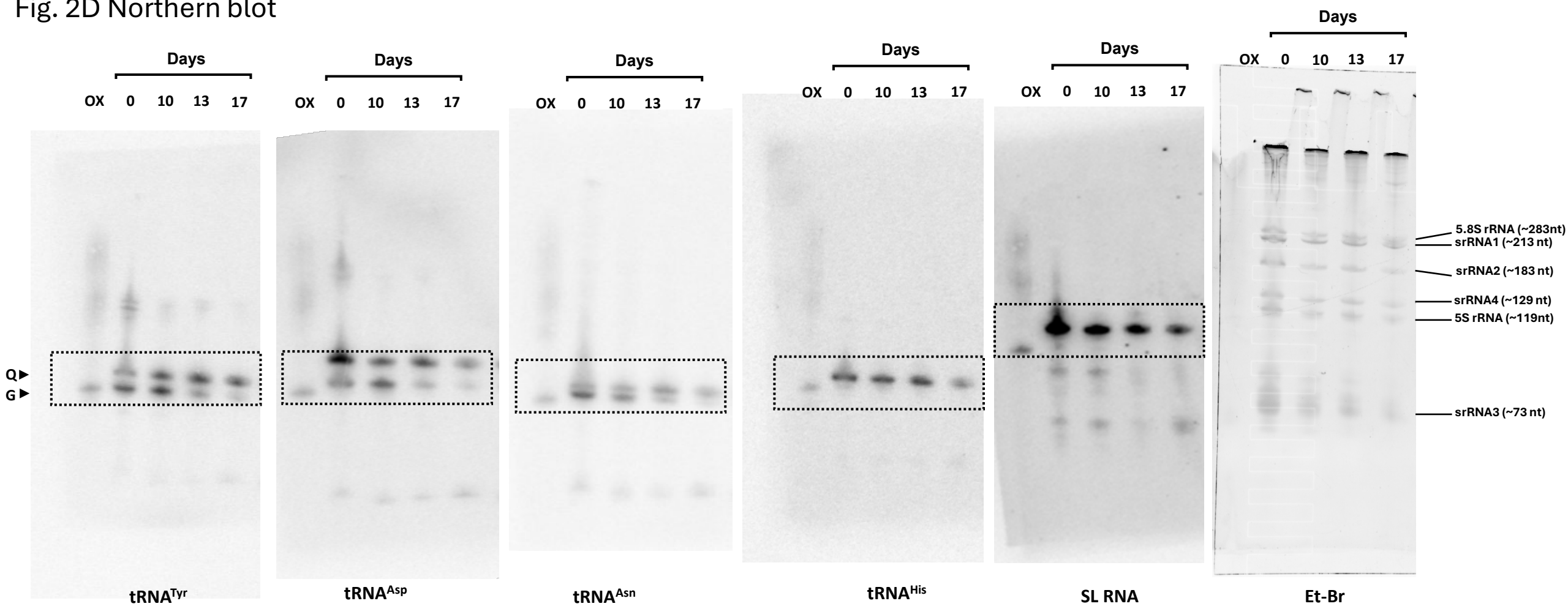

Fig. S1B Agarose gel

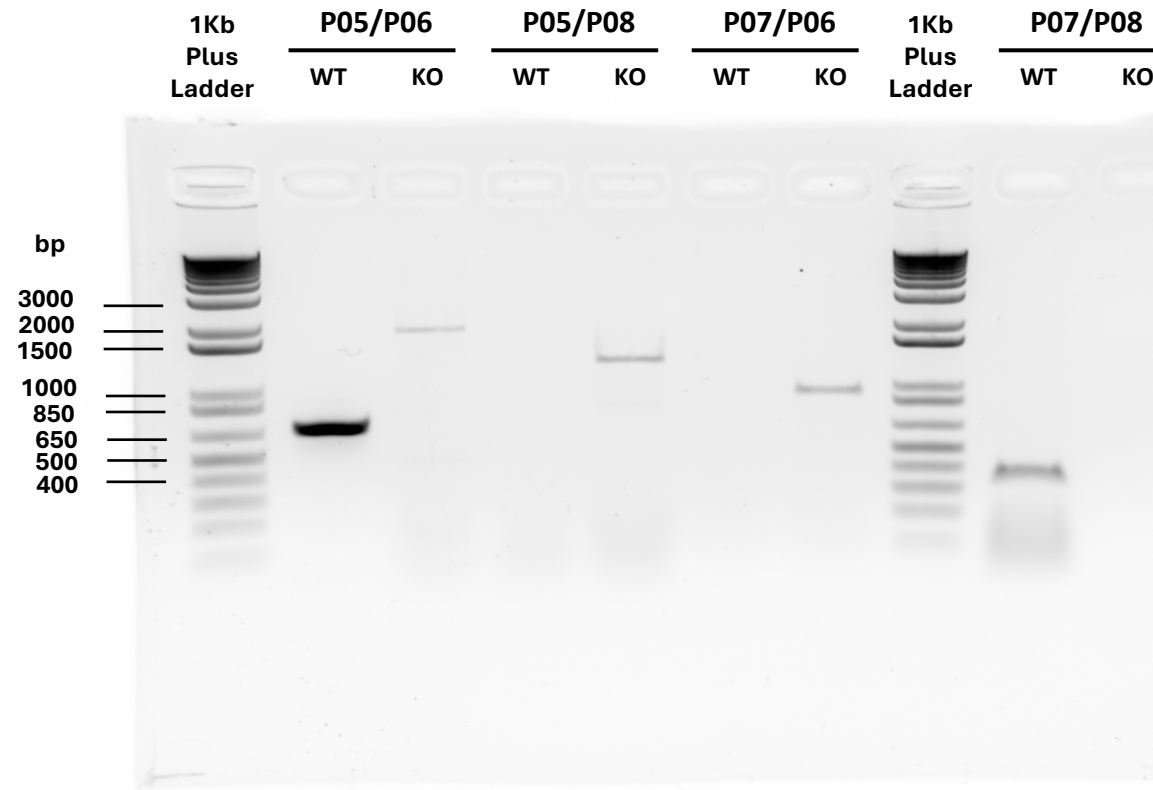

Supplement: S1 Raw Images — (PDF) [file pbio.3003887.s009.pdf]
